# Pharmabiome analyses in tandem with chemometrics can help trace the provenance of falsified medicines: A proof-of-concept study

**DOI:** 10.1016/j.fsigen.2025.103392

**Published:** 2025-11-20

**Authors:** Carla Perez-Mon, Alberto Roncone, Aiman Abrahim, Marivil Islam, Cathrin Hauk, Celine Caillet, Hamid A. Merchant, Rabia Farzand, Luana Bontempo, Simon D. Kelly, Daniel Blessborn, Joel Tarning, Rachel Kline, Victoria Nicheva, Dominic T. Kurian, Paul N. Newton, Rob Ogden

**Affiliations:** a Royal (Dick) School of Veterinary Studies and the Roslin Institute, University of Edinburgh, Midlothian, UK; b Traceability Unit, Research and Innovation Center, Fondazione Edmund Mach, San Michele all’Adige, Italy; c Food Safety and Control Laboratory, Joint FAO/ IAEA Centre of Nuclear Techniques in Food and Agriculture, Department of Nuclear Sciences and Applications, International Atomic Energy Agency, Vienna International Centre, P.O. Box 100, Vienna 1400, Austria; d Medicine Quality Research Group, Centre for Tropical Medicine and Global Health, Nuffield Department of Medicine, University of Oxford, OX3 7LJ, UK; e Infectious Diseases Data Observatory, Centre for Tropical Medicine and Global Health, Nuffield Department of Medicine, University of Oxford, OX3 7LJ, UK; f Mahidol-Oxford Tropical Medicine Research Unit (MORU), Faculty of Tropical Medicine, Mahidol University, Bangkok 10400, Thailand; g Department for Pharmacy, School of Applied Sciences, University of Huddersfield, Queensgate, Huddersfield HD1 3DH, UK; h Department of Bioscience, School of Health, Sport and Bioscience, University of East London, Water Lane, E15 4LZ, UK; i Fera Science Ltd., York BioTech Campus, Sand Hutton, York YO41 1LZ, UK

**Keywords:** Falsified medicines, EDNA sequencing, Mass spectrometry, Stable isotope analyse, Traceability, Origin

## Abstract

A lack of robust analytical approaches limits our ability to investigate the clandestine manufacturing origins of falsified medicines. We conducted a proof-of-concept study to test the feasibility of geolocating the production sites of falsified medicines, based on the identification of site-specific biological and chemo-isotopic features using a combination of environmental DNA metabarcoding, Direct Analysis in Real Time - Mass Spectrometry and Isotope Ratio Mass Spectrometry as profiling techniques. We produced tablets at two distant locations (England vs. Thailand), using controlled manufacturing methods, excipient composition and environmental conditions. Sets of tablets produced at separate locations showed distinct bacterial and eukaryotic diversity, particularly influenced by the incorporation of water used during tableting and the background environmental biosignatures of the production site. Tablets showed corresponding site-specific chemometric profiles, but the factors contributing to the observed chemical differences were unclear. When reference samples of known origin are available, our study suggests that site-specific biological and chemical features can be used in modelling approaches to successfully predict product origin. We developed a new mapping approach to exploit the geographic information within the eukaryotic pharmabiome of the falsifications; based on eDNA-derived species identification and the integration of publicly available species distribution data. In the absence of reference samples of known origin, the application of this workflow to our dataset provided partial clues about the product’s origin, with limitations likely due to taxonomic resolution and the presence of species with wide distribution ranges. Collectively, our research provides experimental support for the development of integrated, multifaceted tools for tracing the origin of falsified medicines, advancing efforts to combat this pervasive but neglected global health problem.

## 1 Introduction

The trafficking of falsified medicines - including vaccines - is a worldwide, yet still largely overlooked, public health threat, and a criminal activity with an annual illegal trade value of 70–200 billion USD [36,59]. Falsified medicines (FMs) are products that fraudulently misrepresent their identity, composition or source [59]. FMs do not contain active pharmaceutical ingredients (APIs), or they contain incorrect amount of the APIs or different APIs to those declared in the packaging (e.g. paracetamol instead of an antibiotic) [63]. Moreover, the ingredients and any API (if present) may not be of pharmaceutical grade nor fit-for-use in medicinal products; often containing impurities or related substances injurious to health. The consumption of FMs by patients can lead to delayed or non-recovery from treatable diseases, and to the emergence of antimicrobial resistance in the case of falsified antibiotics and antimalarials [6]. FMs exacerbate mortality and morbidity rates, mistrust in healthcare systems, and cause economic losses in affected populations [42,58]. These malicious products are particularly prevalent in low- and middle-income countries (LMICs), where studies estimate that FMs, together with substandard medicines (i.e., poor-quality products of non-criminal intention), could account for about 10.5–14 % of the medicines in the market [42,59]. In high-income countries, FMs often enter the supply chains through e-commerce [12], and falsifications of highly valued and demanded products (e.g. SARS-CoV-2 vaccines) have been recently reported [53].

FMs are clandestinely manufactured by cryptic criminal networks or individuals, and smuggled into pharmaceutical supply chains [11,35]. Pharmaceutical supply chains, in turn, can be highly complex and globalized, with the same batch of medicines purchased and distributed in distant locations around the world [60]. Forensic approaches exist to detect medicine falsifications [15,57], yet little research has been done on methodologies to inform where -or from whom- they originate. This lack of traceability is a major obstacle in eradicating FMs; because their production persists even if their local trade is interrupted.

FMs are often produced in unsanitary facilities, using untreated water and excipients of unknown identity [63]. FMs might accrue bacterial and eukaryotic contaminants, also refer as pharmabiome, in the form of environmental DNA (eDNA) from the facility, or the tap water and other ingredients (i.e. excipients) used in their manufacture [62]. In parallel, FMs’ chemo-isotopic features might reflect the raw materials used, whose chemistry can be modified by the manufacturing process and specific environmental conditions at the production sites [49]. Hence, taken together, FMs may contain unique biological and chemo-isotopic signatures, which could indicate their provenance [49, 61,62].

Tablet formulations (e.g. blister-packaged or bottled tablets) are among the most commonly reported FMs [59]. Pilot studies have explored the use of high-throughput eDNA sequencing, chemometric and isotopic techniques to profile FM tablets and produce origin-relevant information [3,16,21,25,39,62]. Young et al. [62] conducted an eDNA metabarcoding investigation on falsified antimalarial tablets collected on Southeast Asia. Based on 16S rRNA and 18S rRNA sequencing, the authors observed that FM tablets that shared a similar type of falsified packaging also shared similar pharmabiomes, which supported the reasoning that biosignatures accrued in the products reflected their provenance. DNA-based taxonomic assignment revealed the presence of plants (e.g., walnuts and hickory species within the Juglandaceae family) commonly found in latitudes around southern China; a region where the tablet’s manufacturing sites were suspected to be located [40]. An earlier study, also on falsified antimalarial tablets investigated the use of Direct Analysis in Real Time - Mass Spectrometry (DART-MS) as a rapid screening tool [3]. The authors were able to discriminate distinct groups of falsified tablets based on their chemical profiles, where group differences seemed to be caused by distinct manufacturing procedures and formulation (“recipe”) changes (i.e., APIs replaced by sugars or by mixes of chloramphenicol and ciprofloxacin in varying proportions). Complementarily, Newton et al. [39] demonstrated the use of Isotope Ratio Mass Spectrometry (IRMS) to differentiate antimalarials via their bulk isotopic composition (*δ*^13^C, *δ*^15^N and *δ*^18^O), whereas the study of Gilevska et al. [21] on ibuprofen demonstrated links between multi-isotope fingerprinting (*δ*^2^H, *δ*^13^C and *δ*^18^O) and product’s country of origin.

Although studies demonstrate that FMs can be differentiated based on their biological and chemo-isotopic features and that geographical signals can be identified, linking FMs to production locations is currently hampered by the lack of understanding of the relative contributions of the manufacturing components (i.e., local environment, raw materials and manufacturing process) to the observed analytical signatures. In the case of tablets, they are often produced using plant-based excipients (e. g., cellulose and starch) imported from third countries, whose biological and chemical signatures could confound those coming from the manufacturing site’s local environment. Likewise, tablets are produced following diverse manufacturing methods, commonly direct compression or wet granulation, whose influences on the chemo-isotopic- and biological- fingerprints of the finished products are not understood. Lastly, product provenance assignment in forensic investigations often relies on comparisons against reference databases comprising samples of known origin, which are largely lacking for FMs; a key limitation in the interpretation of results [11,28]. eDNA-based identification of specific taxa with known, narrow geographic distributions could be a promising approach for reference-free product geolocation, but their utility in tracing FMs has not been thoroughly investigated. Along the pathway to develop analytical tools to trace FM provenance, three crucial questions still need to be answered: (i) *to what extent can biological and chemo-isotopic signatures preserved in FMs be attributed to the local manufacturing environment?* (ii) *Can these signatures assist in geolocating production sites in conjunction with reference samples of known origin? (iii) Can eDNA based taxonomic identification inform geolocation if reference samples are unavailable?*

To address these questions, we conducted a feasibility study in which we compared tablets manufactured under controlled conditions at two distant geographic locations: (a) a laboratory in England and (b) a laboratory in Thailand. At each location, tablets were manufactured using identical formulations simulating three different scenarios of falsification with increasing levels of environmental contamination: (1) by direct -compression in a closed laboratory (2) by direct -compression in an open windows laboratory and (3) by wet granulation using tap water in an open windows laboratory. Tablets were produced without API and using identical excipients of known geographic origin. The tablets were biologically profiled using eDNA metabarcoding and short-amplicon taxonomic markers known to amplify well in trace forensic samples (16S for bacteria, 18S for eukaryotes and trnL for plants) [10,18,33,62]. We also characterised the chemical composition of the tablets and excipients using DART-MS, and bulk *δ*^13^C, *δ*^18^O and *δ*^2^H isotopic composition via IRMS. Our objectives were to:

- Assess whether tablets manufactured in different locations showed distinctive eDNA and chemo-isotopic profiles
- Quantify the contribution of environmental factors vs. excipients to the tablet’s profiles
- Evaluate whether discriminatory eDNA and chemo-isotopic signatures can predict tablet origin, presuming the availability of reference tablets of known origin
- Evaluate whether eDNA-based identification of taxa enables geographic origin assignment of tablets, presuming that reference tablets of known origin are not available

## 2 Materials and methods

### 2.1 Experimental design

Simulated falsified tablets were manufactured in a laboratory in Thailand (Bangkok; 13.76638, 100.53383) and in a laboratory in England (Huddersfield; 53.64307, −1.77759). To produce the tablets, the excipients cellulose (Avicel PH-101 and PH-102 from DuPont Nutrition, in Newark, USA), pregelatinized corn starch (NATIONAL™ 78–1551 from Ingredion, in Indianapolis, USA), corn starch (Purity 21 A from Ingredion, in Rayong, Thailand) and magnesium stearate (KEMILUB EM-F-V from Italmatch Chemicals, in Zuera, Spain) were used. For each excipient, an identical lot -coming from the same batch and container-was divided into two parts: one part was sent to the Thai laboratory (T) and the other part was sent to the English (E) laboratory (4 excipients x 2 laboratories = 8 aliquots in total). In both laboratories, falsified tablets were produced following two methods: direct compression and wet granulation (Table 1). For the direct compression method, aliquots of the cellulose (Avicel PH-102), corn starch (NATIONAL™ 78–1551) and magnesium stearate were mixed in 84:15.5:0.5 w/w proportions, and the mix was compressed into tablets using an automatic TDP 6 s Desktop tablet press (LFA Tablet presses, Taichung City, Taiwan) in the Thai laboratory, and a manual press (MTCM-1 tablet press, Globe Pharma, New Jersey) in the English laboratory. Direct compression tablets were produced under two different environmental conditions: (a) with the windows of the laboratories closed (sample batches T-1 and E-1; closed atmosphere and (b) with the windows of the laboratory open (T-2 and E-2; open atmosphere). The action of opening the laboratory windows allowed external atmospheric inputs to enter, as illustrated by the increase in particulate matter recorded in the Thai laboratory (Figure S1). For the wet granulation method, aliquots of the cellulose (Avicel PH-101) and corn starch (Purity 21 A) were mixed in 84:14 w/w proportions. The mix was then granulated using the local laboratory tap water, oven-dried at 48°C for 10 h (final water content of ~6 %), lubricated with magnesium stearate (2 % w/w) and finally compressed into tablets using the above stated presses at each site (T-3 and E-3). Two additional control groups of wet granulation tablets were produced in the English laboratory: (i) tablets that did not contain water (E-4) and (ii) tablets in which tap water from Bangkok was used instead of the local English water (E-5). These groups were added to better account for the influences of *formulation* and *water* in observed differences between different tablets. All wet granulation tablets were produced with the windows of the laboratory open. A total of 8 groups of tablets were produced (3 in Thailand, 5 in England, Table 1). Laboratory-grade gloves were used in all steps of tablet manufacturing. Tablets were stored at 4°C for DNA testing and at room temperature for chemoisotopic measurements.

### 2.2 eDNA extraction, amplification and sequencing

DNA extractions were performed in the historic DNA facility of the Royal Botanic Garden Edinburgh [17]. DNA Extractions from tablets (3 different tablets per experimental group x 7 groups = 21 tablets in total) were performed on 100 mg of powdered material, using the QIAamp DNA Investigator Kit (QIAGEN, Hilden, Germany) and following to the protocol of Young et al. [62]. Additional DNA extractions were performed on 100 mg of powdered excipients. To account for environmental contributions of the production sites to the tablet’s DNA, settled dust from elevated laboratory surfaces (e.g., tables and windows) was collected as a proxy for atmospheric background [46]. DNA extractions from these samples and tap water controls were performed separately to the test samples. Negative extraction controls (i.e., lysis buffer without sample) were included in each extraction batch. All procedures adhered to forensic-grade standards to minimize external and cross-contamination. Detailed methodologies for sample processing, DNA extraction, and environmental sample collection are provided in the Supplementary Information.

The V4 region of the bacterial 16S rRNA gene and the V9 region of the eukaryotic 18S rRNA gene were amplified via PCR, using the 515 F / 806 R and the Euk1391f / 1510rEukBr universal primer pairs, respectively [62]. The p-loop region of the plastid trnL gene was amplified using the *c-h* primers developed by Taberlet et al. [54]. Additional primers targeting fungi and plants (ITS2 and rbcL) were tested, but failed to amplify in the studied samples. PCRs were carried out in triplicate and negative PCR controls with no DNA template were included to account for background contamination. PCR triplicates were pooled, purified with SPRIselect beads (Beckman Coulter, California, US), and quantified with the Qubit dsDNA HS Assay Kit (Thermo Fisher Scientific, Massachusetts, US). Sequencing libraries were prepared from the purified pools using the NEBNext Ultra II Library Preparation Kit together with the P5/P7 dual barcode indexes (New England Biolabs, Massachusetts, US), according to the manufacturer’s instructions. Libraries were quantified using a D1000 TapeScreen System (Aligent, California, US) and pooled at equimolar concentrations. This final pool was subject to paired-end 2 × 250 bp sequencing on an Illumina MiSeq platform at Edinburgh Genomics (University of Edinburgh, UK). Sequences were demultiplexed by the sequencing facility. See supplementary Information for further details about PCR primers and assays.

### 2.3 eDNA data analyses: sequence processing, biodiversity analyses and predictive modelling

Quality filtering, trimming, merging and amplicon sequence variant (ASVs) identification was performed on the paired-end demultiplexed reads, using QIIME2 2024.2 [5] (Supplementary Information). ASVs with > 10 reads in the negative controls for DNA extraction or PCRs were excluded from downstream analyses. This resulted in final numbers of sequences and ASVs per sample of 5264 - 329,354 reads within 49–790 ASVs for 16S; 23,279–361,675 reads within 62–510 ASVs for 18S; and 9064–170,000 reads within 3–85 ASVs for trnL (Table S1).

ASV diversity and statistical analyses were accomplished using R v4.3.3 [48] within *R Studio* 2024.09.0 GUI [44]. ASV abundance and taxonomic tables generated with QIIME2 were imported into *R* with the package *qiime2R* [4]. Diversity analyses were performed on ASV counts rarefied to 5000 reads through iteration (function rrarefy.perm, *EcolUtils* package [50]. Statistical differences in 16S and 18S α-diversity (richness and Shannon index; not normally-distributed, nor homoscedastic data for all groups) between experimental groups were assessed with Kruskall-Wallis rank-sum tests (kruskal.test function) followed by Dunn post-hoc tests (dunn_test function) in *rstatix* package [31]. Group differences in 16S and 18S community structures (β-diversity) were visualized with Principal Coordinate Analyses (PCoAs), built on Bray-Curtis dissimilarity matrices. Permutational Analyses of Variance (PERMANOVAs, 10^5^ permutations, function adonis in the *vegan* package; Oksanen et al. [41]) were performed to identify the experimental factors underlying statistical differences in β-diversity between groups. Multivariate homogeneity of group dispersion was tested (betadisper function in *vegan* package), to account for the potential influence of distinct within-group variances on the PERMANOVA results [1]. A significance level at 0.05 was considered for all statistical analyses. *p*-values in post-hoc tests were adjusted for multiple comparisons using the Holm method [7].

DESeq2 analyses were applied to identify differentially abundant (da) 16S, 18S and trnL ASVs, separately, between England and Thailand tablets. DESeq2 test design was set to control for differences in manufacturing methods (direct-compression or wet-granulation) within countries (~ country of origin + country of origin:manufacturing method). ASV abundances were normalized for sequencing depth across samples using the *poscounts* method [34], given the high prevalence of ASVs with zero counts. For each normalized ASV, log2-fold ratios between England and Thailand samples were calculated as log2 $\left(\frac{\;\text{ASV}\;\text{abundance}\;\text{England}\;\text{samples}\;}{\;\text{ASV}\;\text{abundance}\;\text{Thailand}\;\text{sample}\;}\right)$. Wald tests were applied to detect the differentially abundant ASVs based on ratio values. *P*-values were adjusted for multiple testing using the Benjamini–Hochberg (BH) method with a false discovery rate threshold of 5 %. Additional da-ASVs were identified using ANCOM-BC following a similar design and adjusted parameters as for DESeq2. To focus on abundant phylotypes, analyses only included ASVs for which the sum of the sequences over all samples was ≥ 1000. To minimize the influence of the excipients on the da analyses, all ASVs present in the pure excipient samples were removed from the data prior to testing. Likewise, the tablet control group E5 (made in England with water from Thailand) was not considered in these analyses; to use it as a test group later in the modelling analyses.

Discriminant Analysis of Principal Components (DAPC) was used to build predictive models of country class based on the 16S and 18S da-ASV rarefied counts, using the *dapc* function in the *Adegenet* R package [29]. All tablets except those in the group E-5 were used to build the models and classified as coming from England (E-1 to E-4, 12 samples) or Thailand (T-1 to T3, 9 samples). The number of PCs to build the models was chosen based on visual inspection of the cumulative variance (~90 % retained variance). Models were validated with the leave-one-out-cross validation (LOOVC) method, through a customized *r* function. Class prediction was performed for group E-5 with the *predict. dapc* function.

### 2.4 eDNA data analyses: taxa annotation and distribution maps

Eukaryotic (18S) and plant (trnL) da-ASVs were annotated to taxon within the QIIME2 environment, with the function *qiime feature-classifier blast* (−p-query-cov −0.9, –p-perc-identity 0.97 and 0.99 for 18S and trnL, respectively), using reference datasets of 18S (221,085 sequences, downloaded from the PR2 18S rRNA database v. 5.0.1, Guillou et al. [26]) and trnL (348,971 sequences, downloaded from NCBI) containing taxonomic information. Taxa annotations were also attempted using the *qiime feature-classifier classify-sklearn* function on corresponding 18S and trnL classifiers (Supplementary Information).

18S and trnL da-ASVs among those successfully assigned to taxa were selected and the taxon distribution ranges were investigated. Selected 18S da-ASVs were those that only occurred in at least two samples in which they appeared overabundant (LFC > 0 for England and LFC < 0 for Thailand). Selected trnL da-ASVs were those remaining after removing da-ASVs that were identified as *Zea sp*. (–p-perc-identity 0.90), as they were assumed to derive from the corn starch excipients rather than coming from the tablets’ production environments. For each selected 18S and trnL da-ASVs, only the BLAST hit(s) with the largest percent identity (LPI) value(s) and annotated to the species level were kept. A given ASV was considered to represent only *one species*, when BLAST analysis resulted in one LPI hit, or if it resulted in more than one LPI hit and annotated to the same species. A given ASV was considered to represent a *set of equally possible species* if BLAST analysis resulted in > 1 LPI hit annotated to distinct species (e.g. one ASV that returned 100 % sequence similarity results to Species A and Species B). If for a given country none of their overabundant da-ASVs could be annotated to the species level, then the genus level was considered instead.

Geographic distributions of species assigned to the selected da-ASVs were retrieved from the Global Biodiversity Information Facility (GBIF; https://www.gbif.org/) and Plants of the World Online (POWO; https://powo.science.kew.org/) databases, using the *R* packages *rgbif* (Chamberlain *et al*., 2017) and *rWCVP* [24], respectively. Additional distribution data for fungal taxa were obtained from GlobalFungi [56]. The BLAST-identified species, together with their geographic information, were divided into four groups: non-plant species represented by the selected 18S da-ASVs that were overabundant in England (group 1) or in Thailand (group 2), and plant species represented by the selected da-ASVs trnL that were overabundant in England (group 3) or in Thailand (group 4). For each group, a unified distribution map was created, comprising all distribution information for all identified species within the group. The GBIF and GlobalFungi databases provide information on specific locations (coordinates) where a species has been reported. In the case of the 18S-identified species, the unified distribution map was created by plotting all coordinates of all species within the group (1 or 2) in the same layout. The *ggplot2::stat_bin_hex* function was used to count the number of observations in delimited map areas. Thus, the larger the count value associated with a binned area, the more times a single species or group of species has been reported to occur in that area. The POWO database provides information on the botanical regions where a plant species is known to be present (1) or absent (0). For the plant species, the unified maps were constructed by superimposing the botanical regions of occurrence of each species within a group (3 or 4). In these maps, the numerical values associated with each botanical region represent the number of species that can be found in that region (e. g., 1 if only one species occurs, > 1 if two or more species occur together). A schematic depiction of the process from sequence analysis to taxon mapping in shown in Figure S2. Alternative maps for tablet and excipient production countries were created using taxa represented by ASVs, whose selection was based on rarefied abundances (Supplementary Information).

### 2.5 DART mass spectrometry measurements

All DART Mass Spectrometry measurements were acquired using a DART-SVP ion source (IonSense Inc, Sangus, MS, USA) interfaced to a micrOTOF QII Quadrupole Time-of-Flight mass spectrometer (Bruker Daltonics, Bremen, Germany). Sample preparation and measurements were performed in the Proteomics & Metabolomics Facility at The Roslin Institute (University of Edinburgh, Scotland, UK). For each experimental group, three technical replicates of four different tablets were measured (3 ×4×8 = 96 measurements). Briefly, tablets’ cores were powdered and resuspended in 99 % ethanol and then pipetted as a 5 µl suspension onto the powder-adherent mesh of DART Quickstrips™ (IonSource, Bruker Daltonics). After ethanol evaporation, the Quickstrips™ were introduced to the DART ion source using an automated rail. DART source was operated in positive ion mode and using the optimised parameters of: helium ionization gas (99.997 %) at a flow rate of 3 L/min with nitrogen (99.998 %) as standby gas, DART grid voltage of 600 V, ionisation temperature of 350 °C and linear rail speed of 2 mm/min. The MicrOTOF system was operated in positive-ion mode, acquiring profile MS data in a mass range of 50–800 *m/z*, using oTOF control software. Capillary voltage and nebuliser gas were set to zero and collision cell energy was set at 10 eV. Quadrupole energy at 5 eV with a PrePulse Ion storage of 10 μs was also set. Acquisition parameters were chosen based on internal tests and previous forensic work on falsified medicine identification and batch discrimination [3,8,52].

Before analysing the tablets, the mass spectrometer was calibrated linearly using PEG-200 (Sigma-Aldrich) as a standard and background spectra were recorded using blank DART Quickstrips™. All spectra data preview and integration was achieved by Compass DataAnalysis 4.2 (Bruker Daltonics, Bremen, Germany) and centroided peak lists were exported for downstream analyses.

### 2.6 DART-MS data analyses

Analysis of spectra was performed with the software *Mass Mountaineer* v7.1.17.0 [13]. Background signal was removed from the tablet spectra and intensity values were normalized as relative intensity values. To evaluate chemical differences between tablets, Principal Component Analysis (PCA) and Kernel Discriminant Analysis (KDA) were performed, with the latter considering the eight groups of tablets (i.e., distinct country of origin, manufacturing method and water sources). We first selected a subset of most abundant features (mass tolerance adjusted to 15mmu and intensity values > 10 %) in the whole dataset (188 in total), by performing ANOVAs (p < 0.05) between the tablet groups showing the largest differences in mean intensity values for each feature. We then built the ordinations based on covariance matrices calculated from the intensity values of the selected features.

To evaluate whether a tablet’s chemical features can be used to predict their country of production, additional covariance-based KDA predictive models were built. In this case, samples were labelled as belonging to England (47 measurements from E-1 to E-4 groups) or Thailand (36 measurements from T-1 to T-3) and discriminant features, used in the model, were selected considering only these two groups. Discriminant features were selected among these two groups using the Fisher Ratios methods, where the 95 features with the largest values and fold change > 1 were kept [13]. Model validation was performed with the LOOCV method. The KDA model was applied to the E-5 tablets (made in England with water from Thailand) to predict their country of manufacture. Tentative compound identification of selected peaks was performed using the PubChem database. Results were graphed in *R*.

### 2.7 Isotopic measurements

*δ*^13^C and *δ*^18^O measurements were conducted at the Fondazione Edmund Mach (Italy) and *δ*^2^H measurements were completed at the International Atomic Energy Agency (IAEA, Austria). All measurements were performed in duplicate (2 different tablets x 8 groups = 16 measurements per element). For the *δ*^13^C measurements, 5 mg of tablet material were placed in tin capsules and isotopic ratios were measured in the filled capsules using a Vario Cube IRMS (Elementar Analysensysteme GmbH) as described in Giannioti et al. [19]. Carbon isotope ratios were normalized against the international reference material USGS88 (*δ*^13^C = −16.06 ‰). The standard USGS90 (*δ*^13^C=-13.75 ‰) was used to check instrument performance. For the *δ*^18^O analyses, 0.2 mg of tablet material was weighed in silver capsules and isotopic ratios were measured using a Finnigan DELTA XP IRMS (Thermo Scientific, Bremen, Germany), following pyrolysis in a Thermochemical Conversion Elemental Analyser (TC-EA; Thermo Scientific, Bremen, Germany), as in Giannioti et al. [20]. Oxygen isotope ratios were normalized against the international reference materials USGS90 (*δ*^18^O = 35.9 ‰) and USGS91 (*δ*^18^O =21.13 ‰). In compliance with IUPAC guidelines [55], final isotopic ratios were expressed as *δ values* relative to the international standards of V-PDB (Vienna-Pee Dee Belemnite) for *δ*^1^ ^3^C and VSMOW (Vienna Standard Meteoric Ocean Water) for *δ*^18^O.

The *δ*^2^H measurements were completed on 0.5 mg of sample crimped in a silver foil capsule. Samples were analysed using a TC-EA connected to a Delta V IRMS via a Conflo IV interface (ThermoFisher, Bremen), following the protocol of Kelly et al. [32]. Final *δ*^2^H values were normalized against the IAEA reference materials V-SMOW2 (*δ*^2^H = 0.0 ‰) and SLAP2 (*δ*^2^H = -427.5 ‰). The accuracy of results was monitored by analysing mineral oil reference material NBS22 (*δ*^2^H = -117.2 ‰) and the IAEA standard GISP water (*δ*^2^H = -189.5 ‰).

## 3 Results

### 3.1 Influences of country of origin and manufacturing conditions on eDNA-biodiversity

**Fig. 1:**
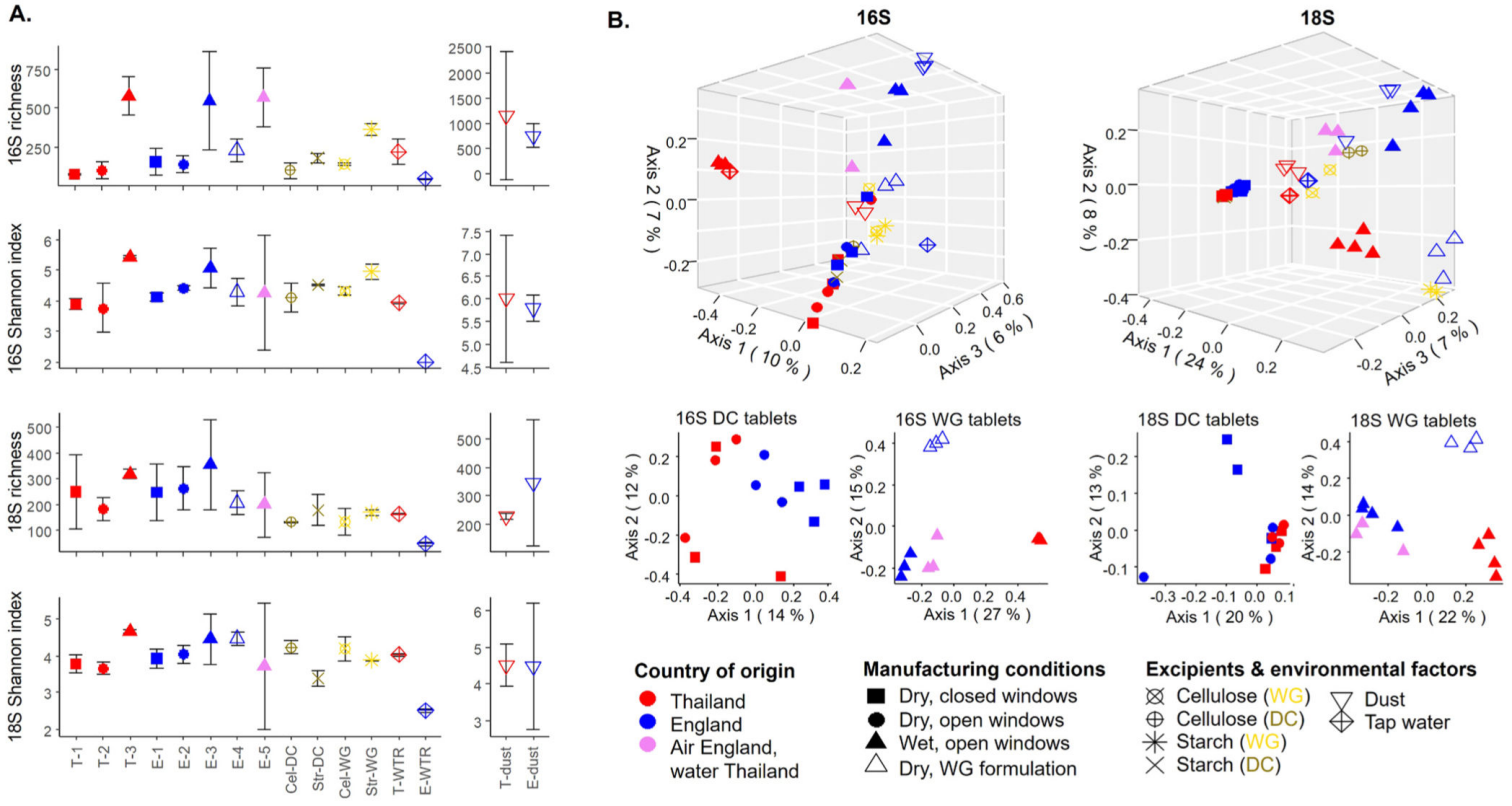
16S and 18S biodiversity of tablets. A. α-diversity. Dots represent the mean ± SD of the richness and Shannon index. B. β-diversity including tablets, excipients and environmental samples (upper panel), or tablets alone (lower panel). PCoAs were computed on Bray-Curtis dissimilarities based on ASVs rarefied abundances. T: Thailand, E: England. Numbers represent dry compression tablets produced with the windows closed (-1), with the windows open (-2), wet granulation tablets (-3), wet granulation tablets that do not contain water (-4) and wet granulation tablets produced in England using Thai water (-5). Cel: cellulose, Str: starch, WTR: water, DC: direct compression, WG: wet granulation.

To assess the influences of country of origin and manufacturing conditions on the overall biological signatures accrued by the tablets, we sequenced the bacterial (16S) and eukaryotic (18S) communities within the samples. Tablets containing water (T-3, E-3 and E-5) generally showed significantly higher mean values of 16S Richness and Shannon Index (α-diversity) than tablets without water (Fig. 1A, Table S2). Similar trends were observed for 18S α-diversity values, but with minor statistical significance (Fig. 1, Table S2). PCoA analyses showed that 16S and 18S community structures (β-diversity) greatly differed between wet-granulation tablets (WG) produced at the two separate countries, whereas the level of differentiation between direct-compression (DC) tablets of different origin was markedly lower (Fig. 1B). WG tablets produced in England but using the water of Thailand (E-5) tended to cluster with the WG tablets produced in England using English water (E-3) rather than the Thai tablets (T-3). Notably, E-3 and E-5 tablets clustered closely with the surface dust controls collected in England. These observed patterns persisted after removing the ASVs present in the excipients from the tablets, to mainly consider laboratory environmental influences within the samples (Figure S3). PERMANOVA tests confirmed the higher influence of water addition, under open laboratory conditions, as a factor promoting differences in community structures between sample groups (9.71 % of explained variation for 16S, and 17.25 % for 18S, p < 0.001 in all cases, Table 2).

### 3.2 Contribution of environmental factors and excipients to eDNA-biodiversity

**Fig. 2:**
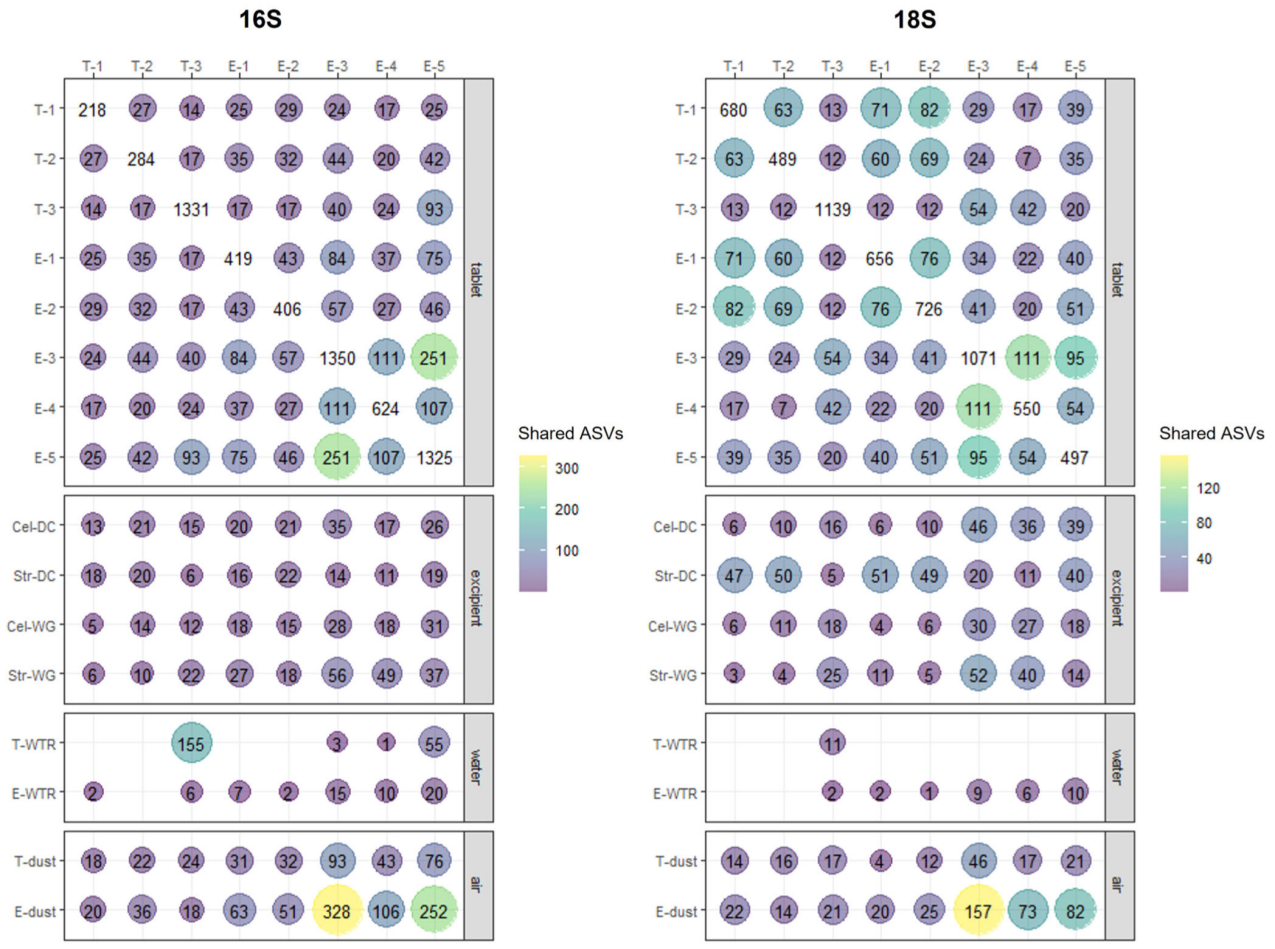
ASVs shared between the tablets, between tablets and excipients, and between tablets and environmental controls (water and air). T: Thailand, E: England. Numbers represent dry compression tablets produced with the windows closed (-1), with the windows open (-2), wet granulation tablets (-3), wet granulation tablets that do not contain water (-4) and wet granulation tablets produced in England using Thai water (-5). Cel: cellulose, Str: starch, WTR: water, Air: environmental dust extracted from swabs, DC: direct compression, WG: wet granulation.

Visualization of 16S and 18S ASVs shared between pairs of tablets, between tablets and excipients and tablets and environmental controls (surface dust and water) showed a high number of ASVs shared between the WG samples and the dust samples, particularly for the tablets manufactured in England (Fig. 2). In line with previous observations, WG tablets produced in Thailand shared the highest number of 16S ASVs with the Thai water. Excipients and DC tablets of distinct origin shared more 18S ASVs than 16S ASVs.

Additional diversity and ASV co-sharing analyses were conducted on the narrower scope of trnL-targeted plant sequences. Unlike for 16S and 18S, plant community structure showed very little differences between tablet origin and manufacturing condition, despite variations in α-diversity (Figure S4, Table S2). Correspondingly, the excipients contributed a larger number of trnL ASVs to the tablets than the surface dust and water controls (0 detected, Figure S5).

### 3.3 Differences in tablet chemo-isotopic profiles associated with country of origin and manufacturing method

**Fig. 3:**
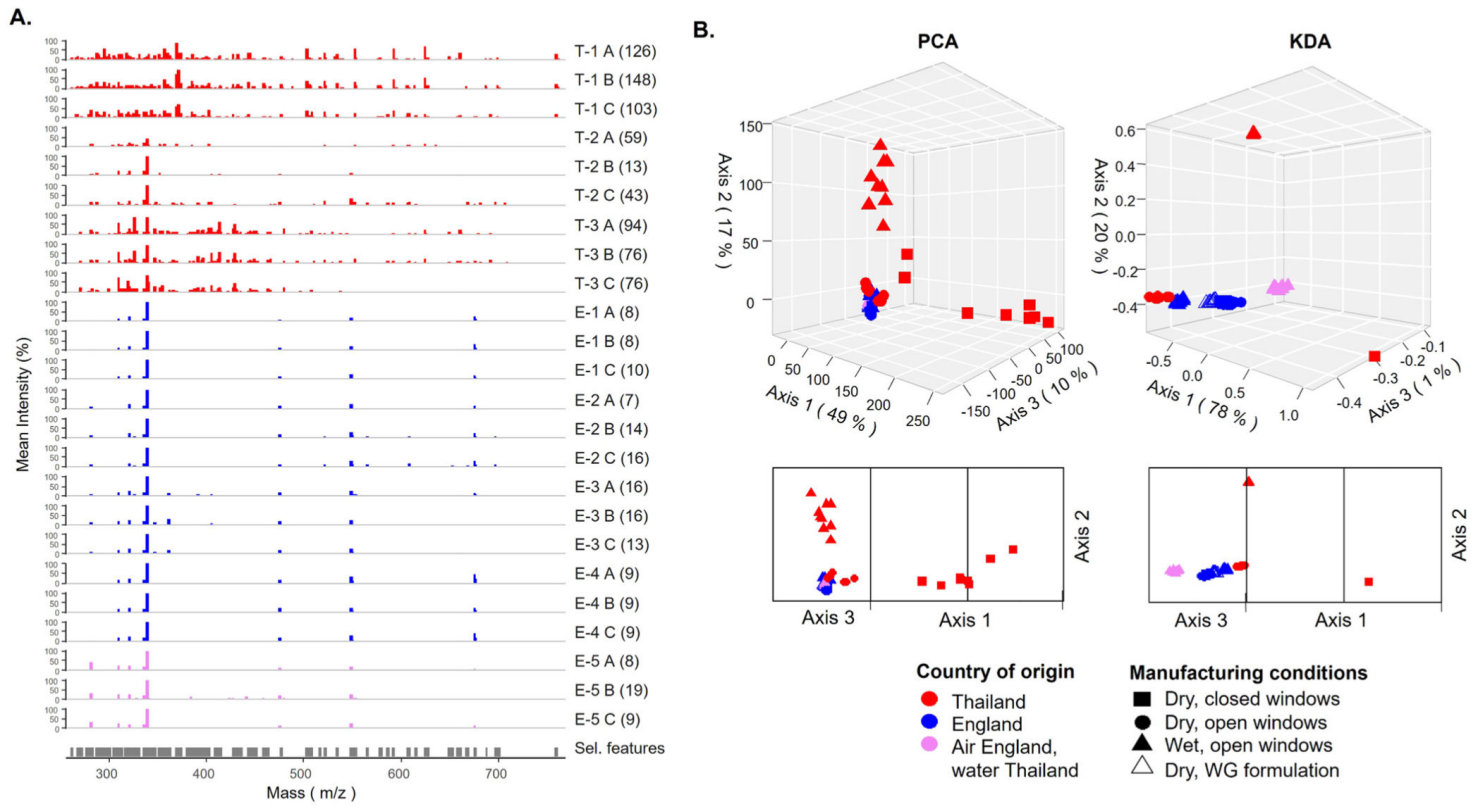
DART-MS profiles of tablets. **A**. Mass spectra of groups of tablets’ groups. Bars represent the mean intensity values of three replicate measurements acquired for a same tablet. Letters represent three distinct tablets (A, B or C) for each tablet group (8 groups in total). Numbers in parentheses represent the total number of masses detected, after aggregating those differing in less than 1 *m/z*. Sel. Features correspond to the subset of masses used for the ordination analyses. **B**. PCA and KDA based on covariance matrices calculated using intensity values of selected features. T: Thailand, E: England. Numbers represent dry compression tablets produced with the windows closed (-1), with the windows open (-2), wet granulation tablets (-3), wet granulation tablets that do not contain water (-4) and wet granulation tablets produced in England using Thai water (-5).

To evaluate the influence of country of origin and manufacture on the chemical and isotopic fingerprint of the tablets we conducted DART-MS analyses and bulk C, O and H isotopic analyses, respectively. DART-MS spectra of tablets produced in Thailand showed a higher number of detected masses (molecular compounds) than tablets produced in England (Kruskal–Wallis test: χ^2^= 11, p < 0.05, Fig. 3A). Unconstrained PCA and constrained KDA ordinations showed that tablets of distinct country of origin clustered separately based on their chemical profiles, with the exception of T-2, whose resemblance to the English tablets was higher than to the Thai samples (Fig. 3B). Manufacturing conditions led to chemometric differences between tablets produced in Thailand, but not between tablets produced in England. Within the English tablets, only minor chemometric differences (along KDA axis 3, comprising 1 % variance) were detected between tablets that did not contain water (E-1, E-2 and E-4) and tablets that contained water (E-3 and E-5). Notably, as with the eDNA results, tablets produced in England using the Thai water (E-5) resembled more closely the samples from England.

The *δ*^13^C, *δ* ^18^O and *δ*^2^H isotopic profiles were similar between all tablets regardless of their country of origin and manufacturing process (Table 3). The *δ*^13^C and *δ* ^18^O, particularly, closely resembled those of the celluloses (84 % content in the tablets), although the inclusion of starch (15 %) shifted the carbon isotopic ratios of the tablets towards less negative values.

### 3.4 Origin assignment based on biological and chemometric profiles

**Fig. 4:**
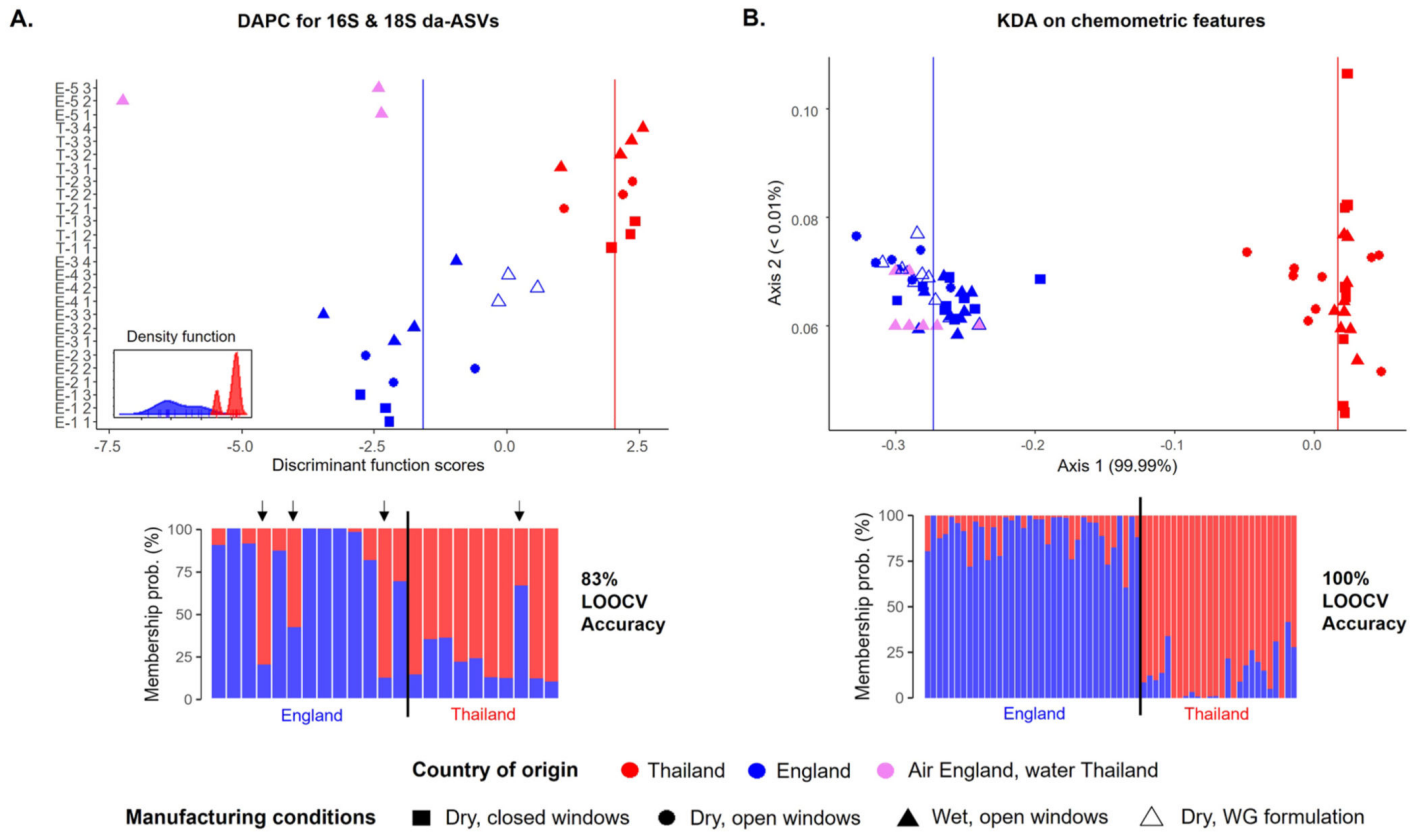
Predictive models for membership assignment of tablets’ country of origin based on **A**. DAPC analyses on the 180 most abundant da-16S and 18S ASVs and **B**. KDA analyses using the 95 most discriminant chemometric features. The upper panel in A. represent each tablet’s scores for the DAPC discriminant function that separates the England vs the Thailand groups, where the blue and red lines represent the mean scores for all the samples belonging to England and Thailand, respectively. The upper panel in B. represent each tablet’s coordinates along the first two axes, where the blue and red lines represent the Axis 1 mean values for all the samples belonging to England and Thailand, respectively. The lower panels show the assigned membership probabilities of the one sample used for testing during the LOOCV of both DAPC and KDA analyses, where arrows indicate misassignments. DAPC included 13 principal components, accounting for 91 % of total variance. Only one DAPC discriminant function was generated because only two groups (England vs Thailand) were defined. Tablets made in England with water from Thailand were not used to build the models, but only in the prediction step. T: Thailand, E: England. Numbers represent dry compression tablets produced with the windows closed (-1), with the windows open (-2), wet granulation tablets (-3), wet granulation tablets that do not contain water (-4) and wet granulation tablets produced in England using Thai water (-5).

We assessed the potential use of the biological and chemometric tablet profiles to predict their place of manufacture; corresponding to an investigative scenario in which reference samples are available from the two alternative possible origins. Discriminant 16S and 18S ASVs between countries were selected through differential abundant (da) analyses (DESEq and ANCOM-BC, p < 0.05, sequences of excipients removed), producing a total of 567 da-ASVs for 16S and 691 da-ASVs for 18S (Tables S3 and S4). DAPC models built on the rarefied counts (> 10 counts across samples) of the selected 16S and 18S da-ASVs were able to classify the tablets to their correct country of origin, with a LOOCV accuracy rate of 83 % (Fig. 4). The 95 most differentiating chemometric features (Fisher Ratios, p < 0.05) between the English and Thai datasets were used to build KDA predictive models. In this case, LOOCV showed that the models were able to fully (100 % accuracy) predict the tablet’s country of origin. In line with previous observations, both sequence-based and chemometric DAPC and KDA models predicted that E-5 tablets (made in England with Thai water, excluded in feature selection and model construction) came from England.

DART-MS features were tentatively annotated to fatty acid amides (i.e. Oleamide, N-Ethyloleamide, Pipericine and Erucamide). Excipients could not be identified in the tablets using DART-MS and, in their pure form, only magnesium stearate (annotated as a stearic acid) and starch (at 500°C ionisation temperature) could be detected.

### 3.5 Origin assignment based on taxon identification and distribution mapping

We evaluated the potential of tablet geolocation assignment using the geographic distributions of identified taxa for selected 18S da-ASVs; emulating the investigative scenario in which reference samples of known origin are absent. To expand the search, we also mapped the distribution of plant taxa assigned to trnL da-ASVs (DESEq analyses England vs Thailand, Table S3).

**Fig. 5:**
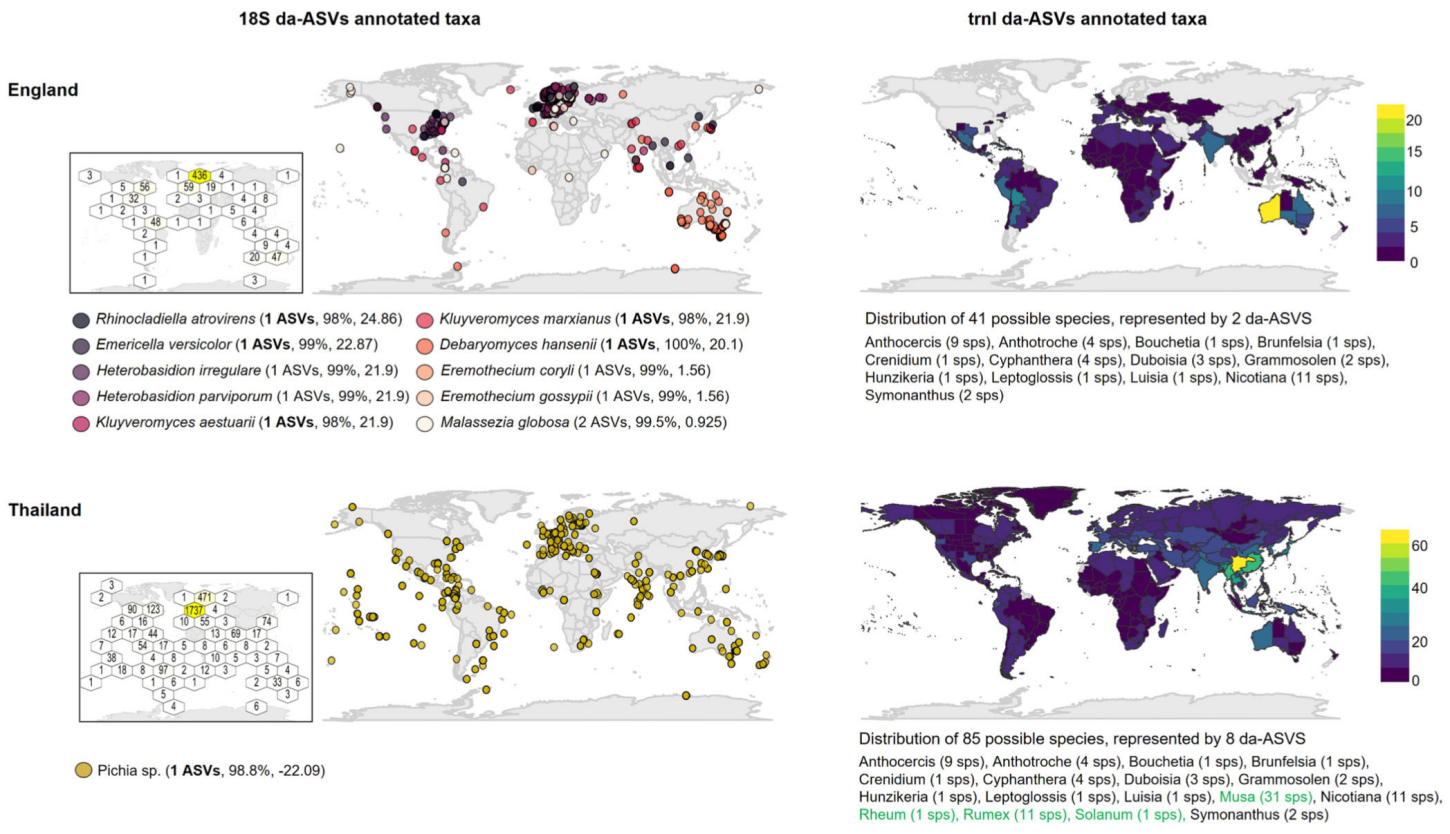
Unified distribution maps for taxa assigned to differential abundant 18S and trnl ASVs, separated by production countries. Distribution information for fungi and plants were obtained from the GBIF and POWO databases, respectively. 18S abundance maps framed in black were built by summing the number of observations (points) in defined map areas, coloured from white (less) to yellow (more) according to the number of observations. trnL maps were built by superimposing the botanical regions in which the distinct taxa are found, coloured according to the number of co-occurring taxa. Bold represent ASVs that exclusively occur in one production country. Green represents plant genera assign to the selected trnl da-ASVs in Thailand but not in England.

Maps showed mixed results, where signals of geographic origin were observed, but unequivocal correspondence could not be established between the taxa’s geographical distribution and the tablet’s production sites (Fig. 5). In the case of tablets produced in England, overabundant 18S da-ASVs were assigned to globally distributed fungal species. GBIF data reported a larger number of observations in northern European regions for the whole set of identified species, but this trend was not observed when using data from the GlobalFungi database (Figure S6). TrnL da-ASVs overabundant in the English tablets were assigned to plant taxa predominantly co-occurring in west Australia. For the tablets produced in Thailand, only one 18S da-ASV, overabundant in these samples, could be confidently assigned below the level of Order, to the fungal genus *Pichia*. GBIF showed a higher number of observations for this taxon in Europe, although the GlobalFungi database reports higher counts in East Asian regions (Figure S7). Plant taxa associated to the Thai samples mostly co-occurred in Asian regions, proximal to Thailand (Fig. 5). Importantly, trnL showed a particularly low taxonomic resolution, where each ASV blasted to numerous species (e.g., ASV sequences blasting to > 10 distinct species with equal percentage identity, Table S5). Likewise, genera largely overlapped between the English and Thai samples.

Similar results to the ones described above were obtained when the most abundant ASVs associated with production country were analysed, regardless of their detection in da-tests (Figures S8 and S9). We also did not observe a clear relation between species distributions and excipient origin (Figures S10-S14). Alternative taxon assignment using 18S and trnL classifiers instead of BLAST analysis only classified a few ASVs to genera and species with broad distribution ranges (Tables S6 and S7).

## 4 Discussion

### 4.1 FMs harbour biological and chemical identifiers of their manufacturing sites

In the quest to develop traceability tools, biological and chemoisotopic profiling of FMs have been proposed as promising approaches to link products to their clandestine production sites, by capturing contaminants from the local manufacturing environment and chemical fingerprints acquired during production. In this proof-of-concept study we addressed a first fundamental question: *to what extent can biological and chemo-isotopic signatures preserved in FMs be attributed to their site of origin?*

Our results provide direct experimental support to the proposal that origin-specific biological and chemical signatures can be measured and quantified in FMs, and that these can be used to discriminate between production sites. More importantly, our results suggest that this site identity can prevail over the signatures contributed by the excipients, which, in real-case FMs can be imported from third country locations and thereby confound geolocation. FM production varies in terms of sophistication, from crude falsifications manufactured in highly unhygienic facilities [37,47] to products generated in relatively sanitary laboratories using specialized equipment and personnel (https://www.painnewsnetwork.org/stories/2019/2/26/counterfeit-pill-lab-exposed-in-bbc-report-nbsp). Likewise, falsifiers might use untreated or treated water sources, presumably of local origin, in products like the wet-granulation tablets tested here, or components such as diluents used in vaccine and liquid medicine falsifications [2]. All these factors might result in FMs containing varying loads of atmospheric or water-associated biological contamination that shape the linkages between a product and its production site.

In our open atmosphere, water-free experiments we did not observe significant alterations or increases in FMs biodiversity, possibly because the sole treatment of opening the windows was not disruptive enough of the otherwise aseptic conditions of our manufacturing laboratories. We did, however, recover a strong biological signal when sanitation conditions were further decreased, by introducing untreated tap water into the falsifications. Strikingly, under these polluted conditions our results suggested a larger contribution of the surface dust biota to the FM fingerprint than that of the water biota itself. Forensic studies addressing trace DNA collection methods have shown a higher DNA uptake when using (dna-free) moist materials compared to dry ones [27,51]. Likewise, investigations on secondary transfers point to higher DNA exchanges between wet samples [23]. Indoor settled dust can be regarded as a composite of airborne, human-shed and outdoors-intrusion materials, which in turn comprises the *local atmospheric background* of our production facilities [46]. It is possible that the addition of water during FMs production, followed by drying, facilitates the deposition of biological detritus coming from this local atmosphere, including eDNA, to the point of overriding the signals exclusively coming from the water. In real-case scenarios, this suggests that even if falsifiers were to use altered or non-local water for their falsifications, the links between products and site of origin could still be preserved. This possibility needs to be more thoroughly explored, e.g., by conducting experiments where FMs are produced using waters of distinct origins and/or with different degrees of sterilization.

Besides site identity, we did not observe clear patterns of association between the simulated FMs chemometric profiles and the tested environmental conditions and manufacturing methods; except for weak evidence suggesting an impact of water source in the chemical signatures. The fatty acid amides identified through DART-MS were not informative of origin, but these compounds could have come from labware materials (or excipients), since they are used as lubricants and slip additives in industrial processes [30]. It is conceivable that other factors not controlled for in this experiment could have driven the origin differences between the MS profiles. For instance, the use of an automatic tablet press in the Thai laboratory (maximum pressure of 60KN, > 10^6^ KPa for tablet die size of 8–10 mm) might have induced a higher fragmentation of excipients than the manual press used in the English laboratory (estimated pressure of 10^5^ KPa); hence explaining the generally richer DART-MS spectra and the high discrimination between batches observed for the Thai samples. We do not however know why the Thai group T-2 resembled more closely the English tablets in the unconstrained ordination analyses. Although we tried to minimise these influences, batch differences between tablets could have also potentially arise from chemical background differences in surfaces, laboratory equipment and tablet’s storage containers at the distinct laboratories.

Concerning real-case falsified medical tablets, variations in machinery and tableting can occur even within a single clandestine facility. High-resolution mass spectrometric techniques might be able to capture these variations and, together with other lines of evidence (e.g., tablet morphometry), could facilitate a better understanding of the clandestine production process. This hypothesis needs however to be tested, e.g., by measuring tablets of distinct origins but similar morphology.

As for the applicability of bulk *δ*^13^C, *δ* ^18^O and *δ*^2^H multi-isotopic profiles to trace FMs, our results suggest that this may not be an effective approach to identify site-origin signatures, as bulk profiles seemed to be dominated by excipient composition. Notwithstanding this, real-world FMs of distinct origin are unlikely to contain the same excipients and/or formulation; and thereby bulk multi-isotopic profiling could still be useful to indirectly identify batches sharing similar characteristics as coming from the same source [21].

### 4.2 FM profiles can be used to predict provenance if reference samples are available

In traceability investigations of illicit drugs, wildlife, food fraud and falsified luxury items, robust approaches using predictive modelling (e. g. Discriminant Analyses and Support Vector Machine algorithms) have been developed to source falsifications based on chemical profiling and interrogation against curated reference databases containing samples of known origin [14,22,28]. Comparative methodologies using (non-human) eDNA profiles to successfully link materials to origins have only recently started to be investigated [18,61]. Some studies have explored the use of reference-based approaches to draw linkages between falsified pharmaceuticals [11,14], but data analytical frameworks to inform FM provenance, at least in the public domain, are lacking. Hence, using predictive modelling we addressed a second question: *Can discriminant (bio)-chemical signatures assist in geolocating FM production sites if using reference samples of known origin?*

Our results support the view that reference-based origin prediction could indeed be a promising route to tackle FMs provenance. We successfully identified discriminant biological and chemical features powerful enough to build predictive models that correctly predicted FMs origin with high accuracies, even if our dataset was small and the samples highly homogeneous. We intentionally used DAPC for the modelling of the FM eDNA signatures because this is a well-established technique, extensively used to identify genetic clusters in genomic studies [38]. Similarly, Kernel Discriminant Analysis was selected for the modelling of the chemical data, given its previous efficacy in DART-MS discrimination of forensic samples [9,45]. Follow up investigations using simulated samples of distinct formulation and produced under more realistic environmental conditions (e.g., non-laboratory or rural settings) are needed to refine these modelling approaches and address limitations in predictive power and overfitting.

Our experimental design and analytical workflow correspond to a claim-counterclaim scenario, differentiating samples from two distinct origins—here, England and Thailand. To broaden the applicability of our modelling approach, reference datasets from additional countries (e. g. through industry or academic collaborators able to produce falsifications in a wider range of locations) should be evaluated. Ultimately, these methods should be validated using fully traced, real-world falsifications.

Lastly, it is important to underline that the techniques used here require sophisticated equipment and specialized operators; hence posing accessibility and cost challenges for low- and middle-income countries. For instance, annual maintenance costs of each platform exceed 10,000 USD, with per sample analyses cost of between 5 and 30 USD. Personnel costs are extremely difficult to compare across countries, however the time taken to process samples ranges from less than one day (DART, IRMS) to a minimum of 1 week for eDNA sequencing. As detailed in Perez-Mon et al. [43], cost-effective development and implementation of origin traceability workflows will require collaborations between academia, industry, medicine regulators, and enforcement agencies. Analyses would occur at established partner laboratories, with findings enabling enforcement agencies to develop actionable anti-trafficking strategies and supporting medicine regulators and pharmaceutical companies in improving product traceability systems.

### 4.3 FM geolocation through eDNA taxa identification might be regarded with caution

One of the major challenges in tracing FMs origins is the lack of reference databases that enable comparison of newly confiscated products with samples of known origin. Previous studies have suggested that phylogeographic interrogation of biological taxa identified within FMs could provide geographic clues about their original location [37,40]. Considering the ability of eDNA sequencing to resolve whole-community taxonomic information, it can be reasoned that the more taxa coming from a region that are identified within FMs as environmental contaminants, the stronger the evidence that the product might come from or be linked to that region [33,61]. This led us to address our third question: *Can eDNA based species identification inform FMs geolocation?*

The results of our study call for caution when it comes to the sole use of eDNA-based taxon identification for geolocation. Assuming the scenario in which samples are of completely unknown origin, we developed an analytical workflow that allowed us to exploit the geographic information contained in the total biological community associated with an FM; by first identifying all possible species represented by discriminant genetic features (ASVs) of tablet origin, and then drawing maps integrating their distribution data at the community-level. There was however only partial correspondence between the shared distributions of the taxa recovered from our simulated FMs and their production locations.

Misleading results could have resulted from a combination of factors. First, the use of short, universal taxonomic markers in our meta-barcoding approach designed to maximize the sequencing yield from the FMs, provided poor taxonomic resolution, hence increasing the chances of non-specific species annotations. Second, most of the identified taxa showed broad geographic distributions, in part due to the lack of species-level resolution. Last, our results are likely biased by the unequal representation of species and locations present in genomic databases and observational repositories (i.e., GBIF and GlobalFungi).

Real-case FMs might contain more, less fragmented DNA than our synthesized FMs [25,62]. This could make it possible to overcome technical limitations, such as the use of longer genetic markers or a panel of markers to improve taxonomic resolution, and possibly recover taxa of narrower distribution range. However, the geographic signal of real-case FM biota will also contain the contribution of the excipients, which, unlike in the controlled samples used here, cannot be so easily removed. Moreover, real-case FMs might be manufactured and packaged at different locations, further confounding the possible origin of their biomes [59]. All in all, even with the most accurate taxonomic assignment, geolocation interpretations based on biogeographic data must always be regarded with caution and be considered alongside other lines of evidence.

## 5 Conclusions

Falsified medicine origins and illegal product linkages are poorly understood. Our study provides direct experimental evidence of distinct biological and chemical features that can link an FM product to its manufacturing site and, therefore, supports further exploration of analytical methodologies in modelling-based prediction of FM provenance; particularly in cases with a limited number of alternative origin scenarios. For broader traceability questions we propose a mapping approach to infer the origin of FMs based on the geographic information contained in their pharmabiome as a whole. However, significant challenges remain in balancing DNA barcoding sequence length, taxonomic resolution and biogeographic precision. Successful forensic investigations require the integration of multiple streams of evidence to support findings. Our multidisciplinary study provides a better understanding of the capabilities of combined FM profiling techniques for origin traceability; thereby representing a step forward towards the development of robust analytical frameworks that will help tackle the global pandemic of medicine and vaccine falsifications.

## Supplementary Material

Supplementary data associated with this article can be found in the online version at 10.1016/j.fsigen.2025.103392.

Supplementary Information

Table S1

figure-Table_S2

Table S3

Table S4

Table S5

Table S6

Table S7

## Figures and Tables

**Table 1:** Experimental design of tablet groups.

Group	Country of manufacture	Manufacturing method^1^	Manufacturing conditions	Main excipients^2^	
T-1	Thailand	Direct compression	Windows closed	Cellulose (84 %, USA),^2^	starch (15.5 %, USA)
T-2	Thailand	Direct compression	Windows open	Cellulose (84 %, USA),	starch (15.5 %, USA)
T-3	Thailand	Wet granulation	Water Thailand, Windows open	Cellulose (84 %, USA),	starch (14 %, Thailand)
E-1	England	Direct compression	Windows closed	Cellulose (84 %, USA),	starch (15.5 %, USA)
E-2	England	Direct compression	Windows open	Cellulose (84 %, USA),	starch (15.5 %, USA)
E-3	England	Wet granulation	Water England, Windows open	Cellulose (84 %, USA),	starch (14.5 %, Thailand)
E-4	England	Direct compression^2^	Windows open	Cellulose (84 %, USA),	starch (14 %, Thailand)
E-5	England	Wet granulation	Water Thailand, Windows open	Cellulose (84 %, USA),	starch (14 %, Thailand)

1 Direct compression tablets did not contain water. Wet granulation tablets contained ~ 6 % of added tap water.

2 E-4 tablets were produced by direct compression using the formulation of the wet granulation tablets but without adding water. The relative proportion of the excipients in the tablets and their country of production are represented in brackets. Starch was obtained from corn. All tablets contained minor quantities (0.5 and 2 %) of magnesium stearate produced in Spain.

**Table 2:** Influence of country of origin and manufacturing conditions on the community structures assessed with PERMANOVA. a= 0.05.

		^1^Df		SS		R2		EV [%]		F		*P*			
16S															
Country		1		0.85		0.09		9.47		2.47		0.0001		***	
Windows opening		1		0.59		0.07		6.61		1.72		0.0144		*	
Water addition^2^		1		0.87		0.10		9.71		2.53		0.0001		***	
Excipient formulation		1		0.58		0.06		6.46		1.68		0.0061		**	
Country X Windows opening		1		0.48		0.05		5.37		1.40		0.0473		*	
Country X Water addition		1		0.77		0.09		8.62		2.24		0.0001		***	
Residual		14		4.83		0.54		53.76							
Total		20		8.99		1.00		100.00							
**18S**															
Country		1		0.58		0.07		7.29		3.17		0.0113		*	
Windows opening		1		1.04		0.13		13.04		5.66		0.0004		***	
Water addition		1		1.37		0.17		17.25		7.49		0.0002		***	
Excipient formulation		1		1.42		0.18		17.92		7.78		0.0001		***	
Country X Windows opening		1		0.20		0.03		2.51		1.09		0.3069			
Country X Water addition		1		0.41		0.05		5.14		2.23		0.046		*	
Residual		16		2.93		0.37		36.84							
Total		22		7.95		1.00		100.00							

1 DF: degrees of freedom, SS: sum of squares, R2: r-squared, EV [%]: explained variation (R2*100), F: pseudo-F ratio, *p: p* value. Tests were performed on Bray-Curtis dissimilarity matrices based on 16S and 18S ASVs, rarefied to 5 264 and 12016 sequences, respectively.

2 All water addition experiments were performed with the windows opened.

**Table 3:** Isotopic profiles of tablets and excipients.

	Country	Manufacturing conditions	*δ*^13^C (‰)^1^	*δ*^18^O (‰)	*δ*^2^H (‰)
**Tablets**					
T-1	Thailand	dry compression, windows closed	-22.5	27.9	-73.3
T-2	Thailand	dry compression, windows open	-22.8	28.2	-72.3
T-3	Thailand	wet granulation, water Thailand	-22.8	28.0	-77.6
E-1	England	dry compression, windows closed	-22.7	29.1	-73.1
E-2	England	dry compression, windows open	-22.9	28.3	-70.7
E-3	England	wet granulation, water England	-23.3	28.4	-79.3
E-4	England	wet granulation, no water	-22.9	28.0	-72.1
E-5	England	wet granulation, water Thailand	-23.1	27.7	-72.9
**Excipients & water**					
Cellulose	USA	Used in dry compression tablets	-25.0	28.6	-50.5
Cellulose	USA	Used in wet granulation tablets	-24.6	28.6	-52.3
Starch	USA	Used in dry compression tablets	-11.2	27.6	-5.2
Starch	Thailand	Used in wet granulation tablets	-11.0	23.9	-20.2
Magnesium stearate	Spain	Used in all tablets		9.4	-234.5
Water	Thailand	Used in wet granulation tablets			-58.0
Water	England	Used in wet granulation tablets			-43.0

1 Isotopic values represent the mean of tablet duplicates per experimental group. Measurement uncertainties are 0.4 ‰ for *δ*^13^C, 0.7 ‰ for *δ*^18^O and 3 ‰ for *δ*^2^H.

## Data Availability

Raw sequences were deposited in the NCBI Sequence Read Archive under the BioProject accession identifier PRJNA1229582. R scripts for data analyses are available in https://github.com/carlaperezmon/Perez-Mon_et_al_2025a
