# Supplementary material for: Pharmabiome analyses in tandem with chemometrics can help trace the provenance of falsified medicines: A proof-of-concept study
Source: Forensic Sci Int Genet. Author manuscript; Available in PMC 2026 Feb 1. (PMC7618506; doi:10.1016/j.fsigen.2025.103392)
Supplement: Supplementary Information [file EMS211583-supplement-Supplementary_Information.pdf]

Supplementary Information

Pharmabiome analyses in tandem with chemometrics can help trace the provenance of falsified medicines: a proof-of-concept study

Carla Perez-Mon[1], Alberto Roncone[2], Aiman Abrahim[3], Marivil Islam[3], Cathrin Hauk[4,5,6], Celine Caillet[4,5,6], Hamid A. Merchant[7,8], Rabia Farzand[7], Luana Bontempo[2], Simon D Kelly[3,9], Daniel Blessborn[4,6], Joel Tarning [4,6], Rachel Kline[1], Victoria Nicheva[1], Dominic T. Kurian[1], Paul N. Newton[4,5,6], Rob Ogden[1]

[1]Royal (Dick) School of Veterinary Studies and the Roslin Institute, University of Edinburgh, Midlothian, UK

[2]Traceability Unit, Research and Innovation Center, Fondazione Edmund Mach, San Michele all'Adige, Italy

[3]Food Safety and Control Laboratory, Joint FAO/IAEA Centre of Nuclear Techniques in Food and Agriculture, Department of Nuclear Sciences and Applications, International Atomic Energy Agency, Vienna International Centre, P.O. Box 100, 1400 Vienna, Austria

[4]Medicine Quality Research Group, Centre for Tropical Medicine and Global Health, Nuffield Department of Medicine, University of Oxford, OX3 7LJ, UK

[5]Infectious Diseases Data Observatory, Centre for Tropical Medicine and Global Health, Nuffield Department of Medicine, University of Oxford, OX3 7LJ, UK

[6]Mahidol-Oxford Tropical Medicine Research Unit (MORU), Faculty of Tropical Medicine, Mahidol University, Bangkok, 10400, Thailand

[7]Department for Pharmacy, School of Applied Sciences, University of Huddersfield, Queensgate, Huddersfield HD1 3DH, UK

[8]Department of Bioscience, School of Health, Sport and Bioscience, University of East London Water Lane E15 4LZ, UK

[9]Oritain UK Ltd., 2-7 Clerkenwell Green, London EC1R 0DE, United Kingdom

Methods

Processing of tablets and excipients for DNA extraction

Processing of tablets and DNA extractions were performed inside dedicated UV-sterilised hoods, at two separated rooms, in the historic DNA facility of the Royal Botanic Garden Edinburgh (Ferrari *et al.*, 2023). Tablets were placed individually into sterile Whirl-Pak bags using sterile tweezers within a UV-sterilised hood. Bags were closed and gently pressed with a pestle in a mortar, to powder the tablet inside while avoiding direct contact with mortar and pestle. For each tablet, 100 mg of powder were weighed into a 2 ml DNA-free Eppendorf, using a sterile spatula. In the case of excipients, already in a powder form, 100mg were directly weighed in the Eppendorfs.

Before each sample was processed, the UV hood surfaces, mortar/pestle and weighing scale were sterilized with detergent and DNA Exitus™ (PanReac AppliChem). To avoid cross-contaminations, tweezers and spatulas were replaced between samples, and a fresh aluminium foil sheet was placed inside the hood each time to serve as a disposable surface on which tablets (or excipients) were processed.

Collection of surface dust and water samples

To control for atmospheric background contamination, dust was collected from the work surfaces of the tablet production's laboratories. In the Thai laboratory, dust samples were collected in duplicate from numerous work surface locations (i.e. tables and windows) using sterile cotton swabs. After collection, the part of the stick that was held by the collector was cut and the swabs were packed in sterile 15 mL tubes. Due to logistical reasons, the dust collection in the Thai laboratory was performed on a same day, days after tablet production. In the English laboratory, dust was collected from work surfaces using sterile cotton towels. Towels were packed in laboratory plastic bags. These samples were collected hours before tablet production, in three separate days. For each day, one towel was used to swab all working surfaces, thereby creating a composite sample. Packed swabs and towels from the two laboratories were sent to the University of Edinburgh where they were stored at 4°C until DNA extraction.

To better understand the influence of water in the eDNA signal, control samples of the same tap water that was used for tablet production were collected at both laboratories. In the laboratories, local taps

were opened, and water was allowed to flow for ~ 1 min to flush out the stagnated portions in the pipes. After this procedure, 1 L plastic bottles were rinse few times using fresh water flowing from the tap. After rinsing, fresh water was collected in the bottles. Bottles were sent to the University of Edinburgh. In the Roslin laboratories at the University of Edinburgh, the waters from the different locations were vacuum filtered through sterile 0.22µm Millipore© Steritop© PVDF filters (Merck, Germany), to trap the sample's biomass. Filters were stored at -20°C until DNA extraction. Two filters were collected per bottle (~500ml of water filtered in each).

Processing of surface dust and water samples for DNA extraction

As for the tablets, processing of surface swabs and water filters was conducted under UV hoods, in the historic DNA facility at the Royal Botanic Gardens of Edinburgh. For DNA extraction of the cotton swabs, the sticks were cut from the heads, and the heads were placed in 2ml Eppendorfs. Only one of the swab duplicates collected per surface location was prepared for extraction. The others were kept at -20°C as backups. In the case of the towels, a square of approximately 5x5 cm was cut and placed in 2ml Eppendorfs. The size of the square was chosen so that the amounts of material used for extraction were comparable between the towels and the swabs; hence between laboratories. The uncut portion of the towels were put back in plastic bags and kept at -20°C as backups.

For DNA extraction of the water samples, duplicated filters (2 for England, 2 for Thailand) were each cut in half. For each filter, one of the halves was placed in a 2ml sterile Eppendorf for subsequent extraction, the other was placed in a sterile petri dish and stored again at -20°C (backup). Filter halves were placed inside the Eppendorfs leaving as many areas exposed as possible, to maximise their contact with the lysis buffer. DNA Extractions of the swab, towel and water filters prepared in the Eppendorfs were performed as described in the Main Manuscript. Blanks for the dust (heads and portions of unused cotton swabs and towels) and water samples (unused filters) were included in the DNA extractions. Sequences obtained from these extracts were removed from the dust and water datasets.

Detailed DNA extraction protocol

DNA was extracted from all processed materials was performed largely following the protocol of Young *et al.* (2022) and using the materials of the Qiagen QIAamp DNA Investigator Kit. Briefly, 1000 ul of lysis

buffer (mix of 900ul of ATL buffer, 30ul of 20mg/ml proteinase k, 45ul of 1M DTT and 25ul of 4mg/ml RNase) were added to the tubes containing the tablet aliquots, and the tubes were incubated overnight in a rotator set at 56°C. The resultant lysates were homogenized by adding 1000ul of AL buffer to the tubes and incubating them at 70°C and 900rpm for 10 min. The homogenized lysates were centrifuged (3-5 min at 16000 g/rcf), the supernatants were recovered and 500ul of absolute ethanol were added to the recovered supernatants. The DNA was then isolated, purified and eluted in 60ul of ATE buffer using the QIAamp silica columns, according to the manufacturer's instructions.

PCR amplification conditions

16S rRNA (515F-806R primers; 5'-GTGCCAGCMGCCGCGGTAA-3' and 5'-GGACTACHVGGGTWTCTAAT-3'), 18S rRNA (Euk1391f-1510rEukBr, 5'-GTACACACCGCCCGTC-3' and 5'-CCTTCYGCAGGTTCACCTAC-3') and trnL (c-h primers; 5'-CGAAATCGGTAGACGCTACG-3' and 5'-CCATTGAGTCTCTGCACCTATC-3'). PCR reactions were carried out in 20 µL assays containing 2 µL of DNA template, 0.3 µM of forward and reverse primers, and 1x DreamTaq master mix (Thermo Fisher Scientific, Massachusetts, US). Thermocycling conditions consisted of 3 min at 95°C (initial denaturation), followed by 35 cycles of 30s at 95°C, 30S at 55°C and 1 min at 72°C (denaturation-annealing-extension) for the bacterial and eukaryotic assays, or 40 cycles of 30s at 95°C, 30s at 58°C and 1 min at 72°C for the plant assays, and a final extension of 10 min at 72°C. The DreamTaq polymerase was chosen because of its good performance against PCR inhibitors.

QIIME parameters for processing of sequences

Quality filtering, trimming, merging and amplicon sequence variant (ASVs) identification was performed on the paired-end demultiplexed reads, using QIIME2 2024.2 (Bolyen et al., 2019). Briefly, the qiime cutadapt trim-paired function was used to remove primer sequences from the reads. Then, the qiime dada2 trim-paired function was employed to trim tails (len_f=200, len_r=140 for 16S, len_f=180, len_r=140 for 18S, len_f=140, len_r=120 for trnL) and merge the reads, remove bad quality mergers, identify ASVs and remove chimeras from the retained mergers.

Obtention and curation of trnL reference sequences for taxonomic annotation

The set of trnL reference reads consisted of all 348 971 available sequences in NCBI GenBank (accessed in January 2024) that were found with the query (trnL[All Fields] OR complete genome[All Fields]) AND (plants[filter] AND (chloroplast[filter] OR plastid[filter])). The sequences were downloaded with the

function efetch -db nuccore from NCBI EDirect command-line interface (Kans, 2010-) and imported into QIIME2. The RESCRIPt functions qiime rescript dereplicate and qiime rescript cull-seqs were used to dereplicated and remove homopolymers and degenerated reads, as described in the trnL Rescript tutorial https://forum.qiime2.org/t/using-rescripts-extract-seq-segments-to-extract-reference-sequences-without-pcr-primer-pairs/23618. The quality filtered trnL sequences were linked to species through their accession numbers, using the nucl_gb.accession2taxid.gz files provided by NCBI (https://ftp.ncbi.nih.gov/pub/taxonomy/accession2taxid/). The complete lineage information of identified species was obtained from NCBI using the package TAXONKit (Shen et al., 2021), and trnL reference sequences whose identity did not correspond to a described species were discarded.

Creation of 18S and trnl classifiers for taxa annotation

18S and trnl classifiers were created within the QIIME2 environment using a set of filtered reads, largely as described in the SILVA RESCRIPt tutorial: https://forum.qiime2.org/t/processing-filtering-and-evaluating-the-silva-database-and-other-reference-sequence-data-with-rescript/15494). For 18S, the filtered reads were obtained from the PR2 database sequences, by performing a round of dereplication and quality filtering (i.e. removal of degenerated reads and homopolymers). For trnl, reads matching the PCR forward (5'-CGAAATCGGTAGACGCTACG-3') and reverse (5'-CCATTGAGTCTCTGCACCTATC-3') primer sequences were extracted from the trnl-NCBI downloaded sequences after a first round of dereplication. Three iterations of reads recruitment, dereplication and quality filtering was then performed to expand the pool of filtered sequences, as explained in https://forum.qiime2.org/t/using-rescripts-extract-seq-segments-to-extract-reference-sequences-without-pcr-primer-pairs/23618. The classifiers were then built on the sets of filtered 18S and trnL reference reads using the function *qiime feature-classifier fit-classifier-naive-bayes*. As for trnl, an additional 18S classifier was also created on primer-selected segments, but the classifier performance was not better than when using full-sequences.

Tablets and excipients' ASV selection based on rarefied abundances

Using the matrices of rarefied counts, four groups of selected ASVs were generated, 18S and trnl ASVs that exclusively occurred either in the tablets produced in England (i.e. ASVs associated to England), or the tablets produced in Thailand (i.e. ASVs associated to Thailand). Among the exclusively occurring 18 ASVs, only those that appeared in at least two samples of their associated countries were retained. All of the trnl-ASVs that exclusively occurred in Thailand appeared only in one sample. To minimize the

influence of spurious reads, only ASVs with more than 100 counts across all samples within each group were considered. ASVs present in the excipients were removed from the tablets prior to the selection. Likewise, trnl-ASVs blasting to Zea sp. (90% coverage and identity) were removed from the trnl datasets.

In the case of the excipients, selected 18S and trnl ASVs consisted on the 10 most abundant ones that were found in the four separate groups of celluloses and starches used in the dry-compression or wet-granulation tablets. All groups of selected 18S and trnl ASVs for the tablets and excipients were blasted to species, and unified distribution maps for highest percent similarity species represented by the distinct groups of selected ASVs were created as explained in the main manuscript.

References

Bolyen E & Rideout JR & Dillon MR*, et al.* (2019) Reproducible, interactive, scalable and extensible microbiome data science using QIIME 2 (vol 37, pg 852, 2019). *Nature Biotechnology* **37**: 1091-1091.

Ferrari G, Esselens L, Hart ML*, et al.* (2023) Developing the Protocol Infrastructure for DNA Sequencing Natural History Collections. *Biodiversity Data Journal* **11**.

Kans J (2010-) Entrez Direct: E-utilities on the Unix Command Line. 2013 Apr 23 [Updated 2023 Dec 6]. In: Entrez Programming Utilities Help [Internet]. ((US) BMNCfBI, ed.) p.^pp. https://www.ncbi.nlm.nih.gov/books/NBK179288/.

Shen W & Ren H (2021) TaxonKit: A practical and efficient NCBI taxonomy toolkit. *Journal of Genetics and Genomics* **48**: 844-850.

Young JM, Liddicoat C, van Dijk KJ, Tabernero P, Caillet C, White NJ, Linacre A, Austin JJ & Newton PN (2022) Environmental DNA as an innovative technique to identify the origins of falsified antimalarial tablets-a pilot study of the pharmabiome. *Scientific Reports* **12**: 21997.

Supplementary figures

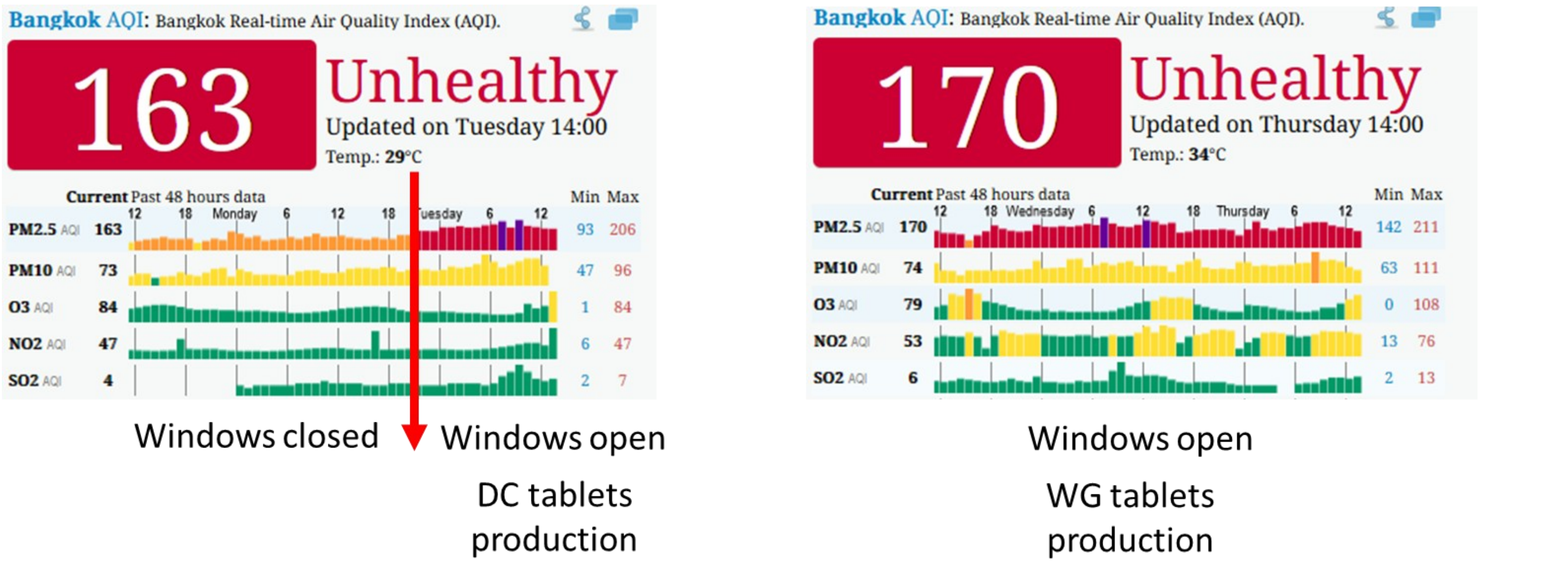

Figure S1. Air quality measurements inside the laboratory in Thailand, before and after opening the windows to produce direct compression (DC) and wet granulation (WG) tablets.

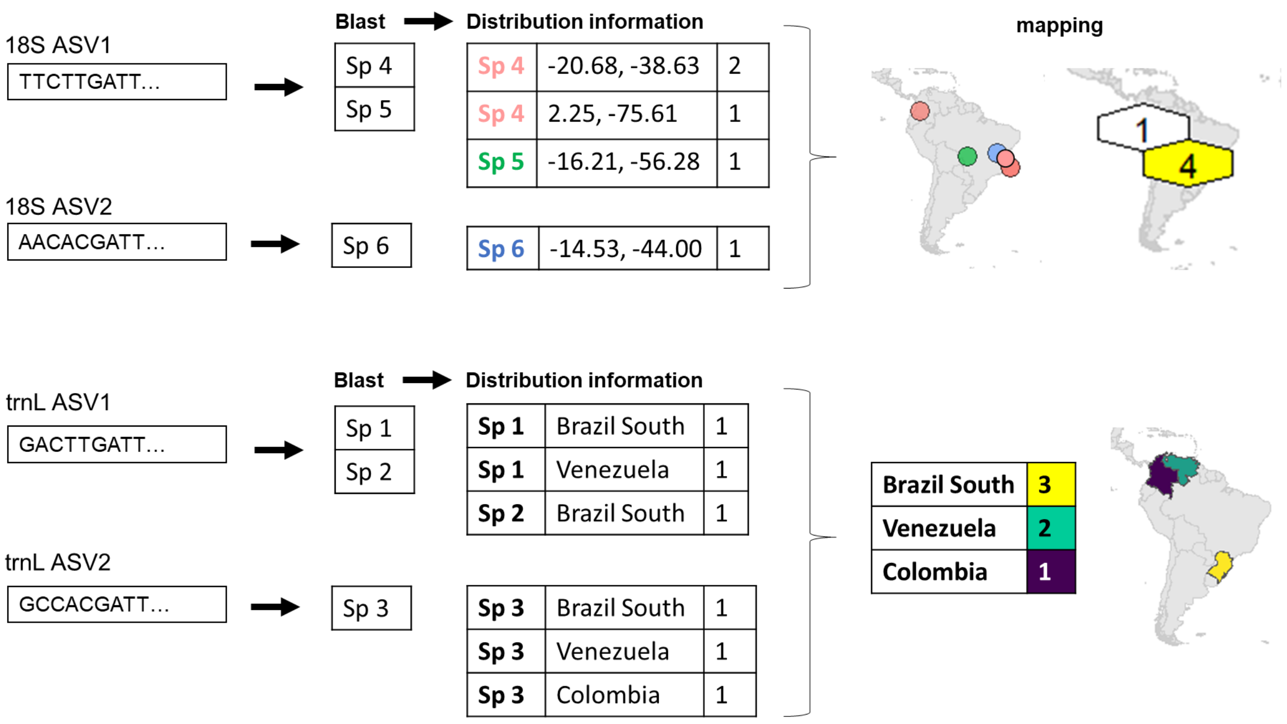

Figure S2. Graphic representation of the process of ASVs taxa assingment, and use of taxa distribution information to produce unified species distribition maps for groups of samples. For each selected ASV per set of samples (e.g. ASVs of higher abundance in tablets produced in England compared to tablets produced in Thailand) a list of species were obtained, of equal (blast) percent identity to the ASVs. For each species in the list, the distribution information was obtained, corresponding to exact locations where the species has been observed in the case of 18S ASVs (GBIF and GlobalFungi data), or to botanical regions where the species can be found in the case of trnl ASVs (POWO data). All colated geographic information for all possible species representing all ASVs in a group of samples (e.g. tablets produced in England) were plotted in one map. For 18S ASVs, coordinates corresponding to observed species were plotted all together as points on the map, or points/obervations were aggregated as the sum of all observations for all species in a defined hexagon region. For trnl ASVs, botanical regions of all plant species were superimposed in a same map. A score with an associated color was given for each region, representing the number of species in the list that could be found in that region.

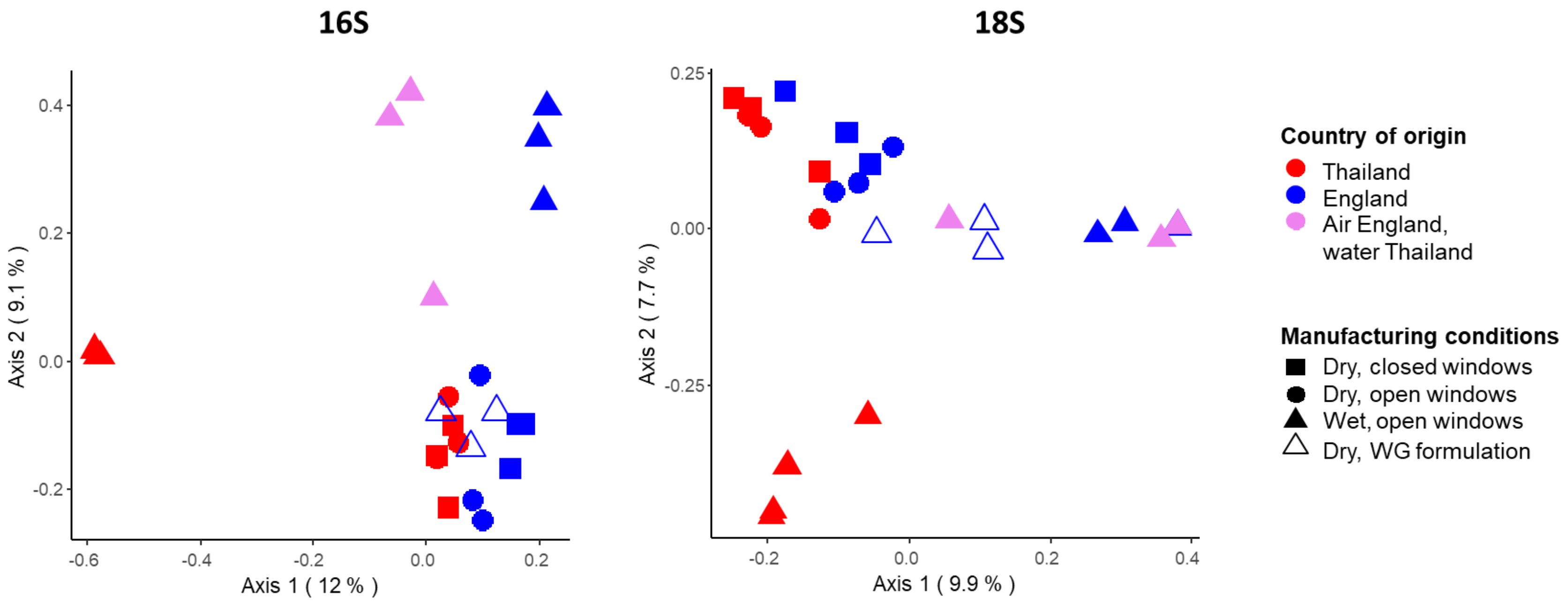

Figure S3: 16S (upper panel) and 18S (lower panel) β-diversity of tablets after excluding ASVs present in the excipients. Excipients ASVs removal was performed as a mean to further evaluate the laboratory environmental influences in the tablets' biomes. WG: wet granulation.

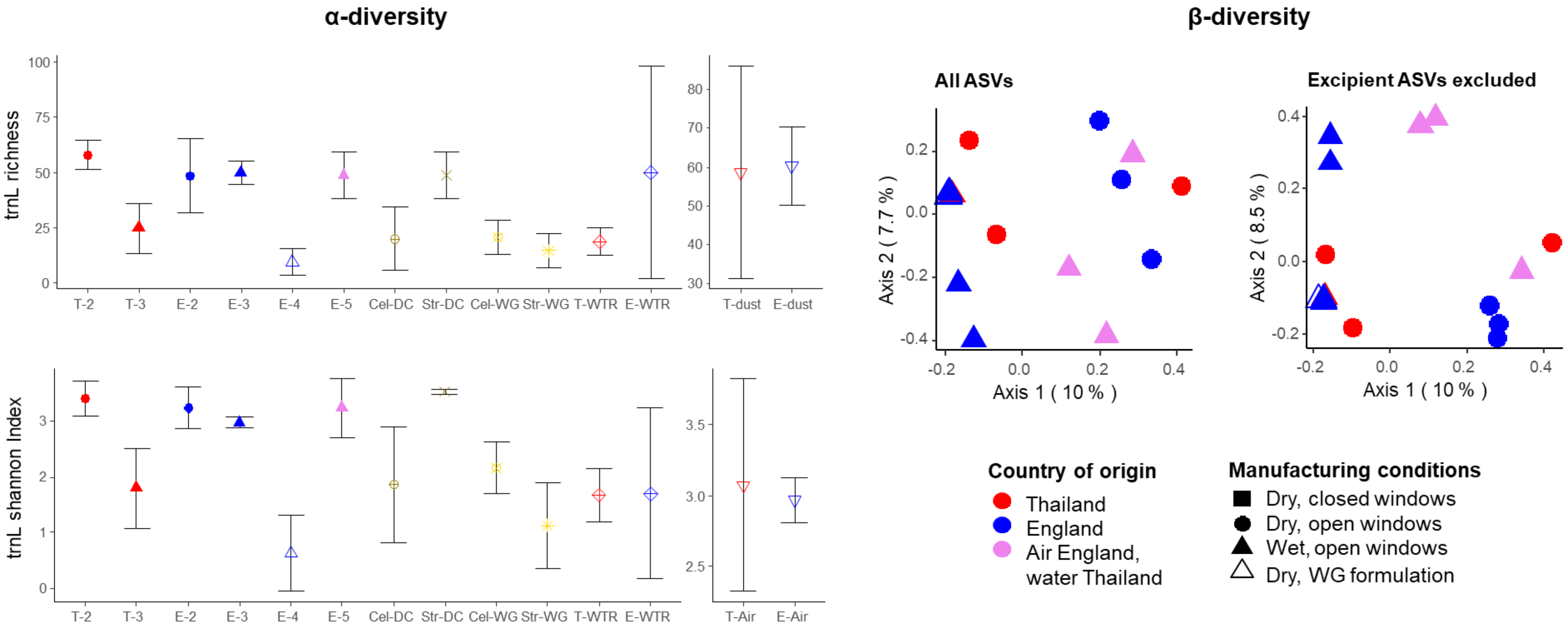

Figure S4: tnrL α- and β-diversity of tablets. Dots represent the mean ± SD of the richness and Shannon index. Tablet β-diversity was calculated both before (all ASVs), and after excluding the ASVs present in the excipients (Excipients ASVs excluded). Excipients ASVs removal was performed as a mean to further evaluate the laboratory environmental influences in the tablets' biomes. T: Thailand, E: England. Numbers represent dry compression tablets produced with the windows open (-1), with the windows closed (-2), wet granulation tablets (-3), wet granulation tablets that do not contain water (-4) and wet granulation tablets produced in England using Thai water (-5). Cel: cellulose, Str: starch, WTR: water, Air: environmental dust extracted from swabs, DC: direct compression, WG: wet granulation.

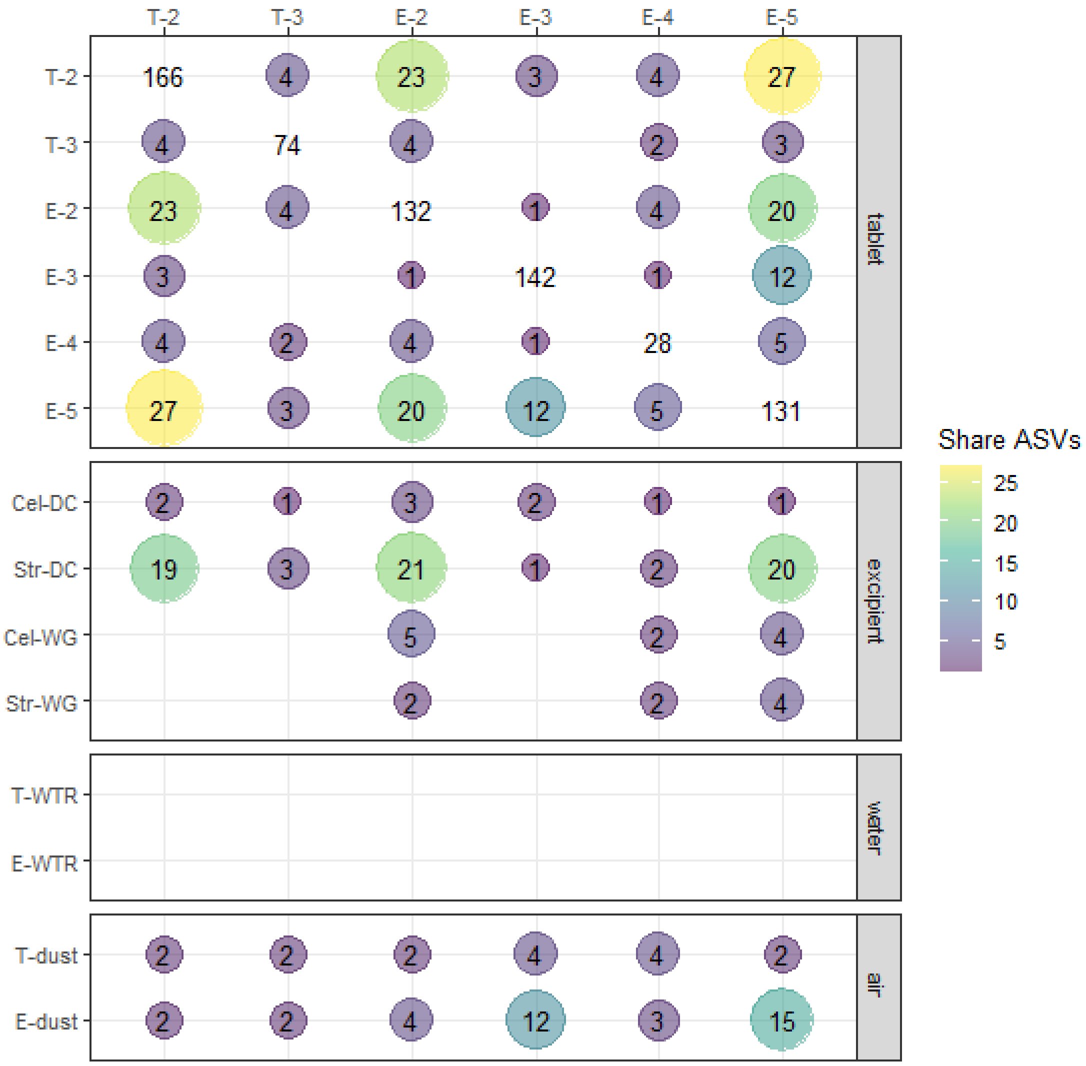

Figure S5. trnL ASVs shared between the tablets, between tablets and excipients, and between tablets and environmental controls (water and air). T: Thailand, E: England. Numbers represent dry compression tablets produced with the windows closed (-1), with the windows open (-2), wet granulation tablets (-3), wet granulation tablets that do not contain water (-4) and wet granulation tablets produced in England using Thai water (-5). Cel: cellulose, Str: starch, WTR: water, Air: environmental dust extracted from swabs, DC: direct compression, WG: wet granulation.

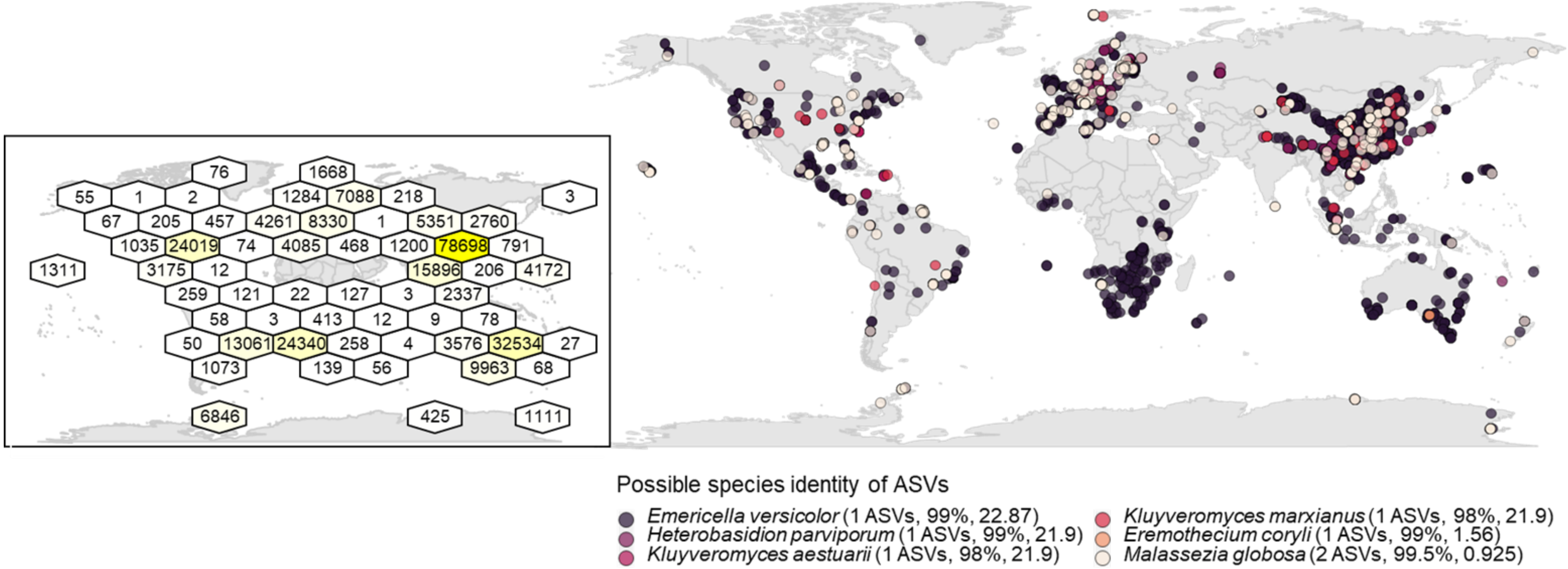

Figure S6: Unified distribution maps based on GlobalFungi database for fungal taxa annotated to 18S da-ASVs overabundant in England. Abundance map framed in black was built by summing the number of observations (points) in defined map areas, coloured from white (less) to yellow (more) according to the number of observations.

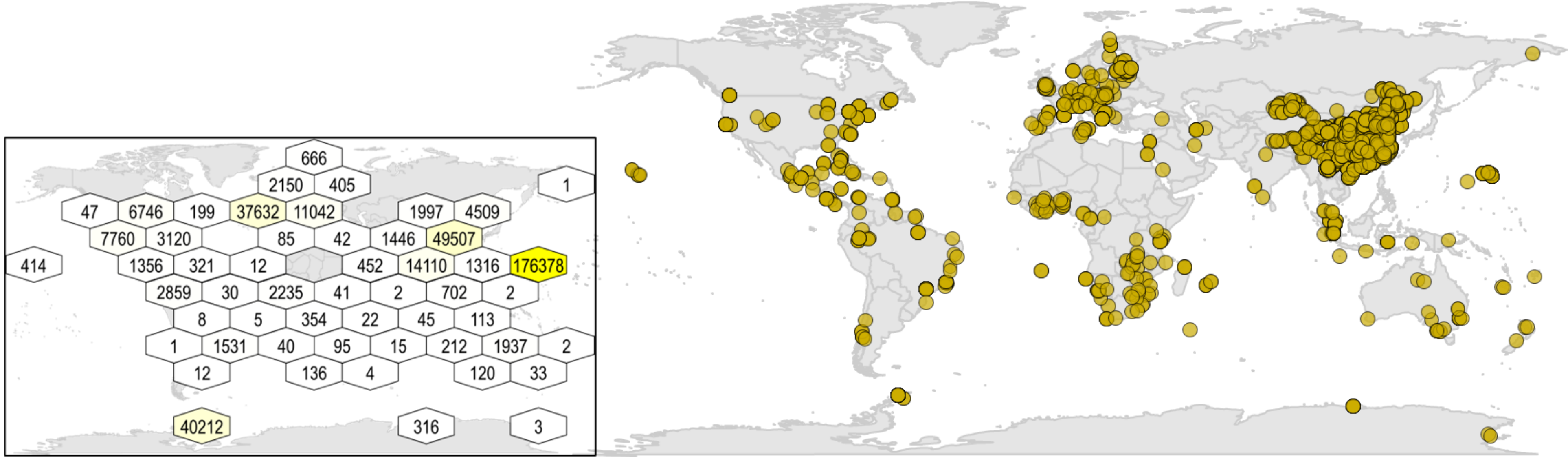

Figure S7: Distribution map of Pichia sp. based on GlobalFungi database. Abundance map framed in black was built by summing the number of observations in defined map areas, coloured from white (less) to yellow (more) according to the number of observations.

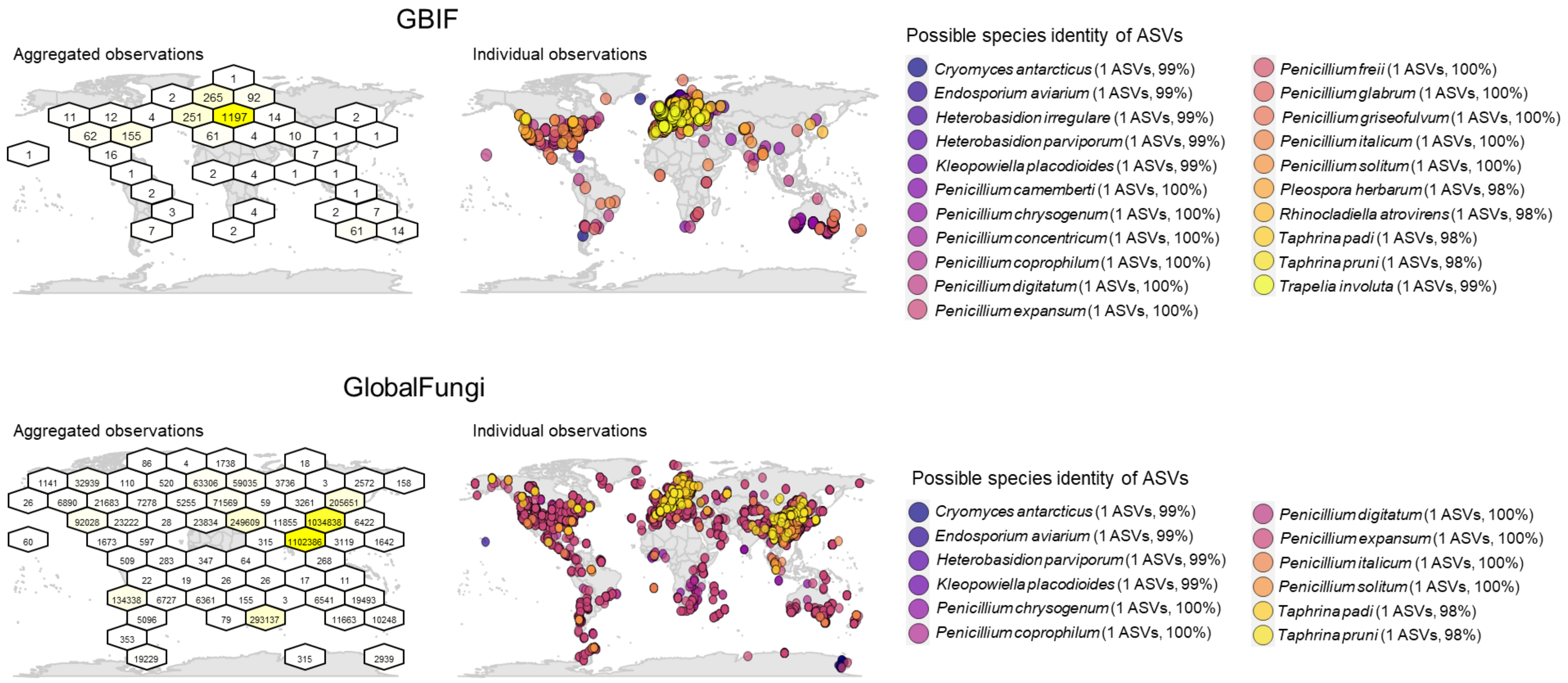

Figure S8: Unified distribution maps for fungal taxa annotated to top-abundant ASVs in the english tablets, based on the information in GBIF and GlobalFungi databases. Abundance map framed in black was built by summing the number of observations in defined map areas, coloured from white (less) to yellow (more) according to the number of observations (aggregated observations). No top-anundance ASVs were identified within the Thai samples that blasted to specific species.

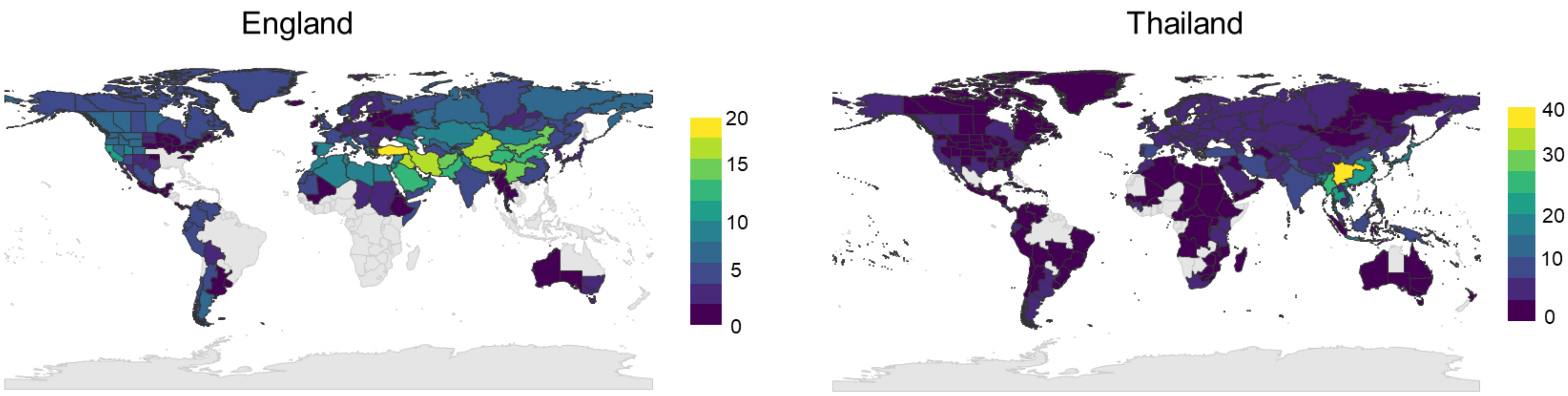

Distribution of 72 possible species, represented by 3 ASV

Alopecurus (2 sps), Amphibromus (4 sps), Anthoxanthum (1 sps), Apera (3 sps), Bellardiochloa (3 sps), Cinna (2 sps), Plantago (6 sps), Poa (51 sps)

Distribution of 43 possible species, represented by 4 ASV

Musa (31 sps), Rheum (1 sps), Rumex (11 sps)

Figure S9: Unified distribution maps for plant taxa annotated to top-abundant ASVs in the english and thai tablets, based on the information in POWO database.

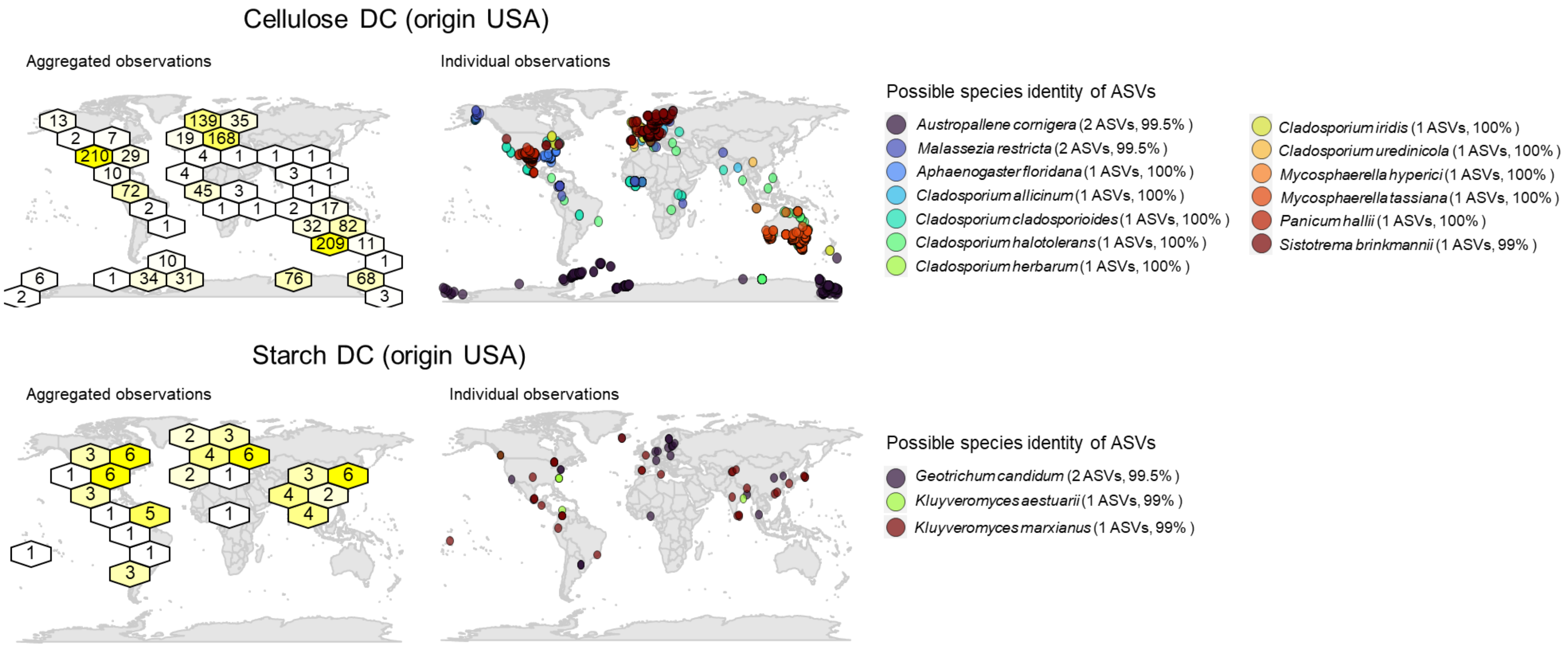

Figure S10: Unified distribution maps for taxa annotated to top-abundant ASVs in the excipients used in dry-compression (DC) tablets, based on the information in GBIF database. Abundance map framed in black was built by summing the number of observations in defined map areas, coloured from white (less) to yellow (more) according to the number of observations (aggregated observations).

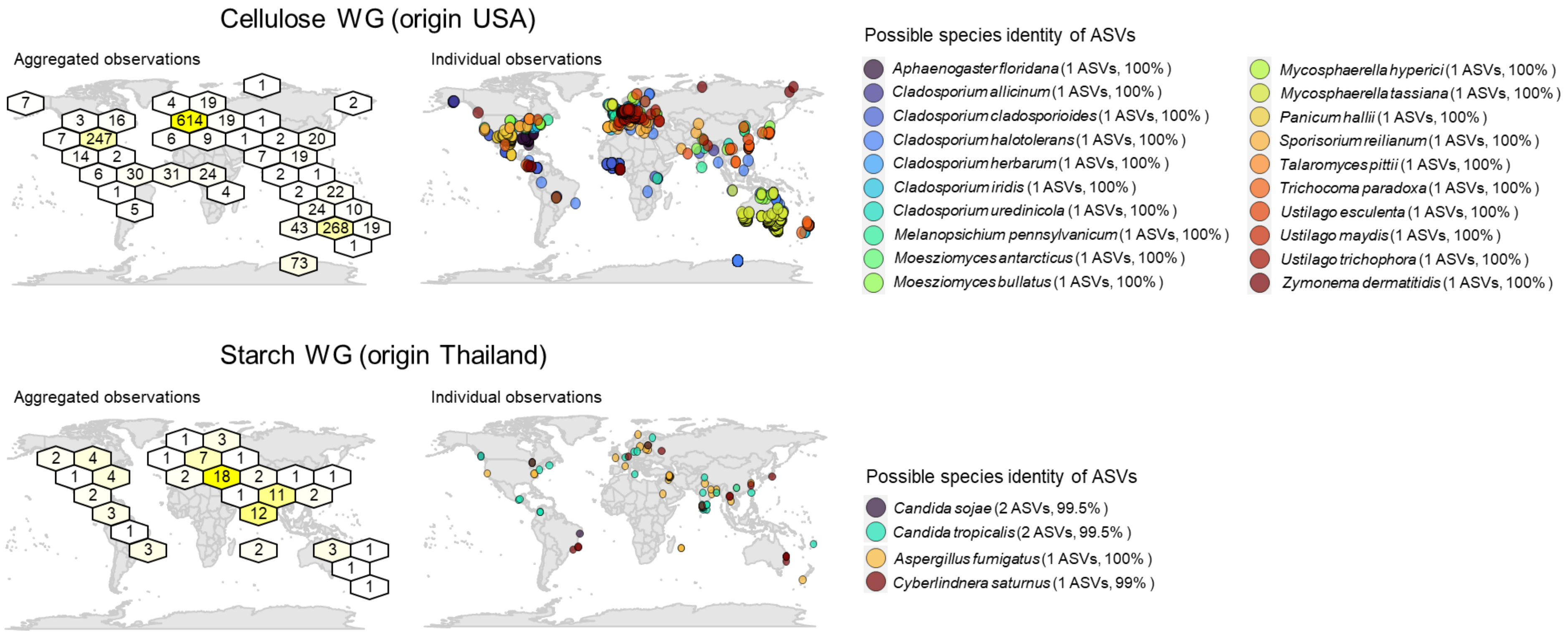

Figure S11: Unified distribution maps for taxa annotated to top-abundant ASVs in the excipients used in wet-granulation (WG) tablets, based on the information in GBIF database. Abundance map framed in black was built by summing the number of observations in defined map areas, coloured from white (less) to yellow (more) according to the number of observations (aggregated observations).

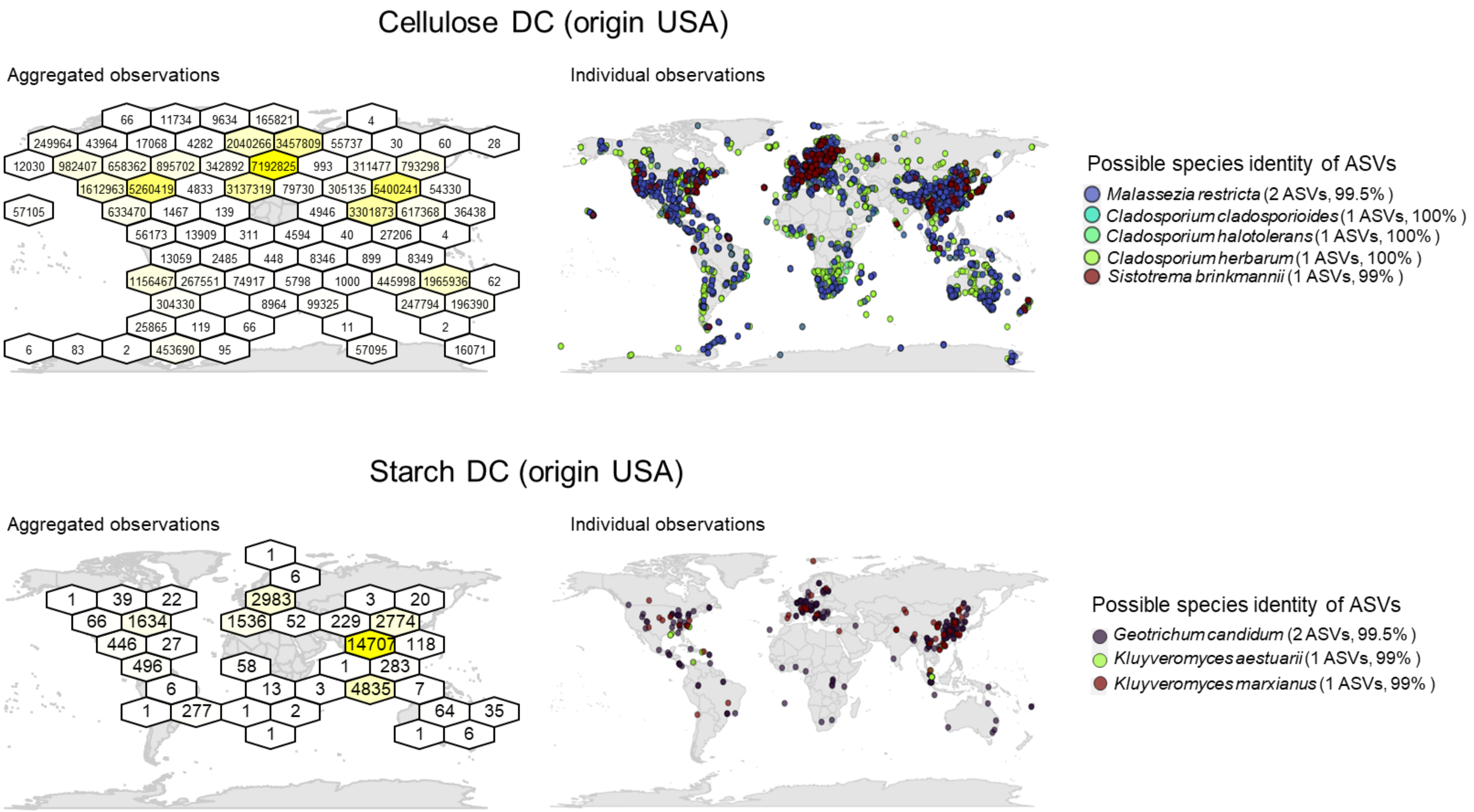

Figure S12: Unified distribution maps for taxa annotated to top-abundant ASVs in the excipients used in dry-compression (DC) tablets, based on the information in GlobalFungi database. Abundance map framed in black was built by summing the number of observations in defined map areas, coloured from white (less) to yellow (more) according to the number of observations (aggregated observations).

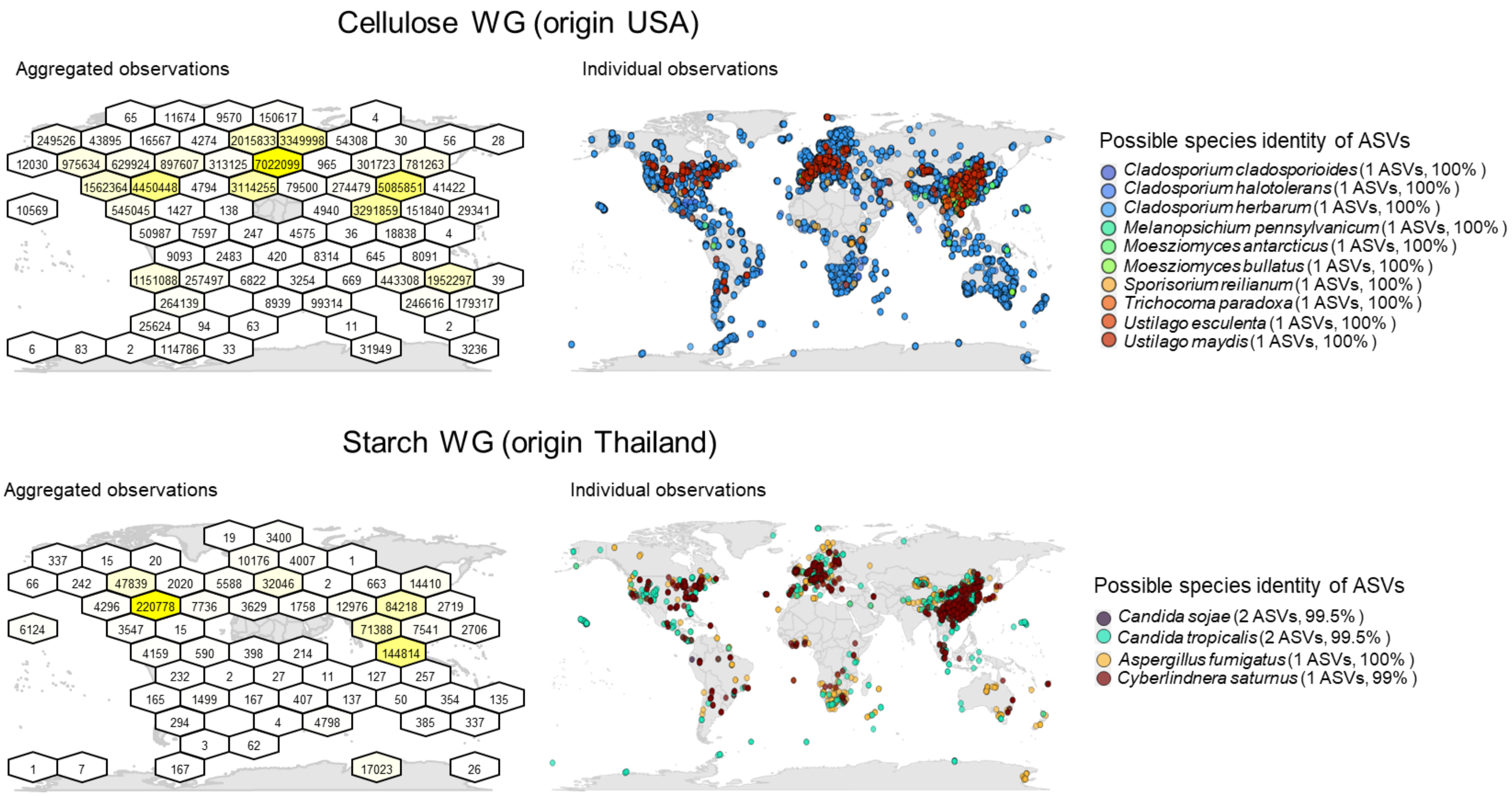

Figure S13: Unified distribution maps for taxa annotated to top-abundant ASVs in the excipients used in wet-granulation (WG) tablets, based on the information in GlobalFungi database. Abundance map framed in black was built by summing the number of observations in defined map areas, coloured from white (less) to yellow (more) according to the number of observations (aggregated observations).

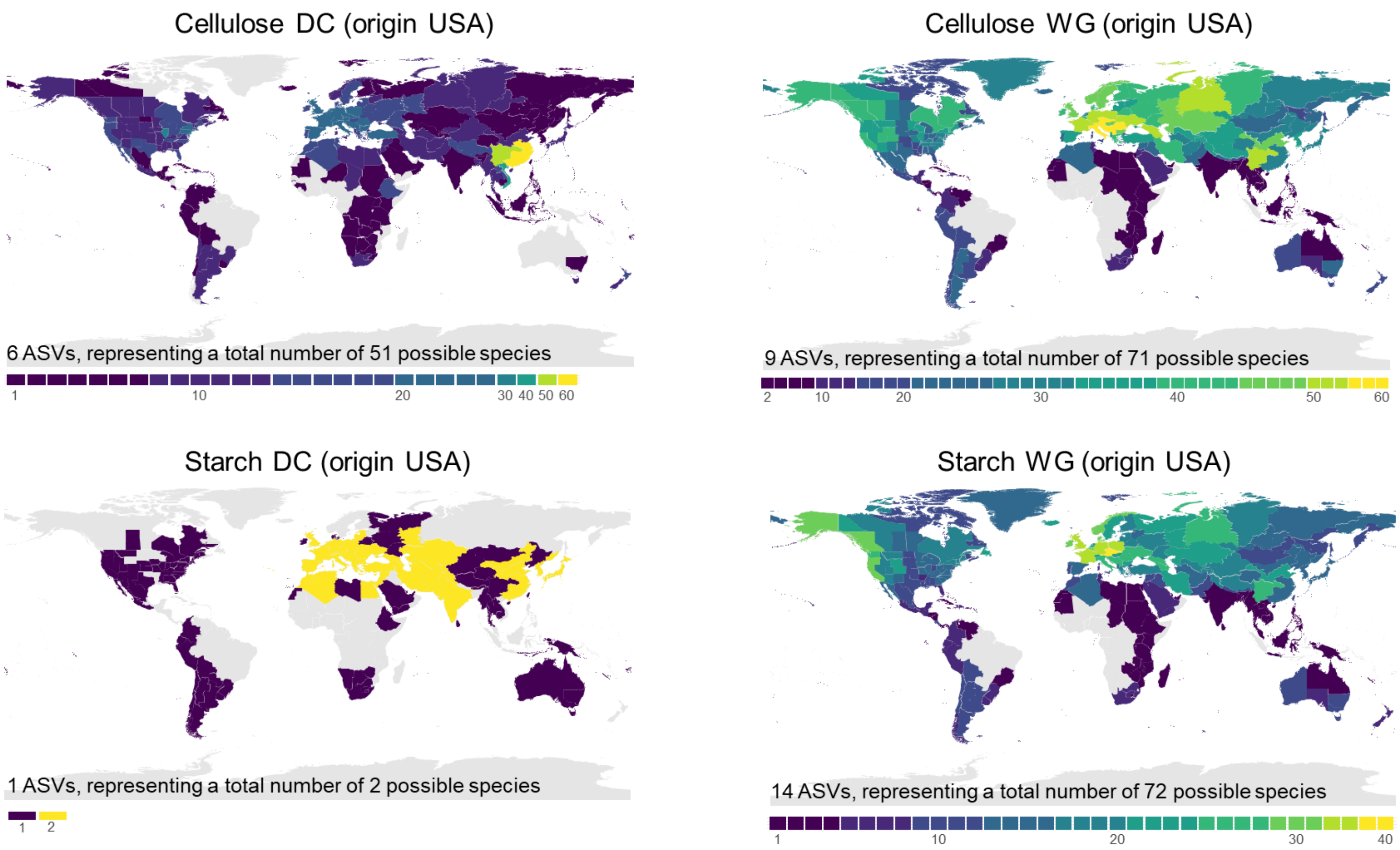

Figure S14: Unified distribution maps for plant taxa annotated to top-abundant ASVs in the excipients used for the dry-compression (DC) and wet-granulation (WG) tablets, based on the information in POWO database.

Supplementary tables

Tables are provided in xlsx.

Table S1: Total and percentage of high-quality sequences and ASVs before and after removing control reads

Table S2: Effects of site and manufacturing conditions in 16S, 18S and trnL α-diversity

Table S3: DESeq2 results for 16S, 18S and trnL ASVs

Table S4: ANCOM-BC results for 16S and 18S ASVs

Table S5: Plant species represented by the ASVs associated to the tablets produced at Thailand and England, and to the excipients

Table S6: 18S-ASVs rarefied abundance and taxa assignment using a customized PR2 classifier

Table S7: trnl-ASVs rarefied abundance and taxa assignment using a customized trnl-NCBI classifier
